# Replisome-cohesin interactions provided by the Tof1-Csm3 and Mrc1 cohesion establishment factors

**DOI:** 10.1007/s00412-023-00797-4

**Published:** 2023-05-11

**Authors:** Sudikchya Shrestha, Masashi Minamino, Zhuo A. Chen, Céline Bouchoux, Juri Rappsilber, Frank Uhlmann

**Affiliations:** 1 Chromosome Segregation Laboratory, The Francis Crick Institute, London, NW1 1AT UK; 2 Bioanalytics Unit, Institute of Biotechnology, Technische Universität Berlin, 13355 Berlin, Germany; 3 Wellcome Centre for Cell Biology, University of Edinburgh, Edinburgh, EH9 3BF UK

**Keywords:** Sister chromatid cohesion, DNA replication, Cohesin, Tof1-Csm3, Mrc1, *S. cerevisiae*

## Abstract

The chromosomal cohesin complex establishes sister chromatid cohesion during S phase, which forms the basis for faithful segregation of DNA replication products during cell divisions. Cohesion establishment is defective in the absence of either of three non-essential *Saccharomyces cerevisiae* replication fork components Tof1-Csm3 and Mrc1. Here, we investigate how these conserved factors contribute to cohesion establishment. Tof1-Csm3 and Mrc1 serve known roles during DNA replication, including replication checkpoint signaling, securing replication fork speed, as well as recruiting topoisomerase I and the histone chaperone FACT. By modulating each of these functions independently, we rule out that one of these known replication roles explains the contribution of Tof1-Csm3 and Mrc1 to cohesion establishment. Instead, using purified components, we reveal direct and multipronged protein interactions of Tof1-Csm3 and Mrc1 with the cohesin complex. Our findings open the possibility that a series of physical interactions between replication fork components and cohesin facilitate successful establishment of sister chromatid cohesion during DNA replication.

**Supplementary Information:**

The online version contains supplementary material available at 10.1007/s00412-023-00797-4.

## Introduction

The monumental effort of DNA replication would be wasted without sister chromatid cohesion, the process that keeps replicated chromosomes together until their timely separation in anaphase. Concomitantly with DNA replication, cohesion between sister chromatids is established by the ring-shaped cohesin complex, which topologically entraps DNA (Nasmyth and Haering 2009; Peters and Nishiyama 2012; Uhlmann 2016). Cohesin molecules are loaded onto DNA during G1 phase of the cell cycle with the help of a cohesin loader complex. The turnover of cohesin on DNA at this point is high, as cohesin’s DNA association is dynamic and subject to the unloading action of Wapl (Gerlich et al. 2006; Lopez-Serra et al. 2013). During S phase, the DNA replication fork inevitably encounters cohesins. However, details of what happens during these encounters remain elusive. Following DNA replication, cohesion between sister chromatids has been established—cohesin now topologically encircles two sister DNAs (Haering et al. 2008). Furthermore, cohesin is acetylated at two conserved lysine residues of its Smc3 subunit by the Eco1 acetyl transferase, stabilizing cohesin’s association with DNA (Ben-Shahar et al. 2008; Ünal et al. 2008; Zhang et al. 2008). Chromosome segregation then occurs after sister chromatid alignment on the mitotic apparatus, when cohesins are proteolytically cleaved by the enzyme separase to trigger anaphase (Uhlmann et al. 2000).

Different scenarios have been contemplated to explain cohesion establishment during replication fork-cohesin encounters. Firstly, G1-loaded cohesin might be converted into cohesive cohesin during replication fork passage. This could be achieved either by replication fork passage through cohesin rings, or through transfer of cohesin behind the fork (Lengronne et al. 2006). In a second model, cohesin is *de novo* loaded behind the replication fork, sequentially entrapping the leading and lagging strands (Murayama et al. 2018). Thirdly, cohesin might be pushed by replication forks to replication termination sites where sister chromatid cohesion is established (Cameron et al. 2022). These models are not mutually exclusive, as genetic experiments have found evidence that more than one parallel cohesion establishment pathway operates in cells (Xu et al. 2007; Borges et al. 2013; Srinivasan et al. 2020).

Several non-essential components of the replisome have been identified as “cohesion establishment factors,” i.e., proteins that enable the establishment of sister chromatid cohesion but are not part of the cohesin complex. These include Ctf4, Chl1, Ctf18-RFC, Tof1-Csm3, and Mrc1 (Hanna et al. 2001; Mayer et al. 2001; Mayer et al. 2004; Skibbens 2004; Xu et al. 2004). Ctf4 serves as a replisome interaction hub that, among other proteins, recruits the Chl1 helicase to contact cohesin during cohesion establishment (Samora et al. 2016). The Ctf18-RFC complex increases PCNA levels at the replication fork, on top of the PCNA that is required for DNA synthesis (Liu et al. 2020). PCNA in turn recruits Eco1 that, depending on transient DNA structures that arise during DNA replication, acetylates Smc3 (Moldovan et al. 2006; Minamino et al. 2023). Tof1-Csm3 and Mrc1 form a heterotrimeric complex that associates with the CMG helicase (Bando et al. 2009; Eickhoff et al. 2019; Baretić et al. 2020). Tof1 and Csm3 intimately interact to form a functional unit, where deletion of either component has the same consequence on sister chromatid cohesion as deleting both. While Tof1-Csm3 further associate with Mrc1, removing Mrc1 results in sister chromatid cohesion defects additional to those due to absence of Tof1-Csm3, suggesting that Mrc1 acts in a parallel cohesion establishment pathway (Xu et al. 2007). It has been suggested that Tof1-Csm3 contributes to converting G1-loaded cohesin into a cohesive form, while Mrc1 supports a de novo cohesin loading pathway (Srinivasan et al. 2020). The mechanisms by which Tof1-Csm3 or Mrc1 perform any of these possible roles are yet unknown and are the subject of our present study.

Tof1-Csm3 and Mrc1 have been studied for their diverse roles during DNA replication. Mrc1, originally identified as mediator of the replication checkpoint, promotes Rad53 checkpoint kinase activation in response to replication stress (Osborn and Elledge 2003; Pardo et al. 2017). Tof1-Csm3 and Mrc1 couple helicase and polymerase progression when forks slow down, and for this role have become known as the “fork protection complex” (Katou et al. 2003). Tof1-Csm3 and Mrc1 also act to secure full replisome speed under undisturbed conditions, leading to their alternative denomination as “replisome progression complex” (Tourriere et al. 2005; Yeeles et al. 2017). Tof1, first known as topoisomerase I interacting factor (Park and Sternglanz 1999), furthermore recruits topoisomerase I to the replisome, a function that may relate to the role of Tof1 in imposing fork pausing at genetically encoded replication fork barriers (Shyian et al. 2020). Tof1 also interacts with the nucleosome chaperone FACT to enhance nucleosome disassembly (Safaric et al. 2022). Additional reported roles for Tof1-Csm3 and Mrc1 include replication through secondary structures and repetitive DNA (Razidlo and Lahue 2008; Gellon et al. 2019; Lerner et al. 2020), as well as replisome disassembly (Deegan et al. 2020).

Here, we investigate whether Tof1-Csm3 and Mrc1 contribute to cohesion establishment through one of their replication functions. By systematically disrupting the replication checkpoint, fork speed control, as well as topoisomerase I and FACT recruitment, we find that the role of Tof1-Csm3 and Mrc1 in sister chromatid cohesion establishment is distinct from these known replication functions. Instead, we find that Tof1-Csm3 and Mrc1 engage in a series of direct physical interactions with the cohesin complex. We arrive at a model in which replisome components not only secure faithful DNA replication, but directly engage with the cohesin complex to link DNA replication with sister chromatid cohesion establishment.

## Results

### The replication checkpoint and sister chromatid cohesion establishment

The replication checkpoint is crucial in moments of replication fork stalling, e.g., at sites of DNA damage or following nucleotide depletion (Pardo et al. 2017). A low level of replication checkpoint activation is detectable even during unperturbed S phase (Forey et al. 2020). It is therefore conceivable that replisome-cohesin encounters lead to temporary checkpoint activation, which might facilitate sister chromatid cohesion establishment. In support of this hypothesis, single molecule observations in *Xenopus* egg extracts revealed frequent fork stalling upon replisome-cohesin encounters (Kanke et al. 2016).

**Fig. 1:**
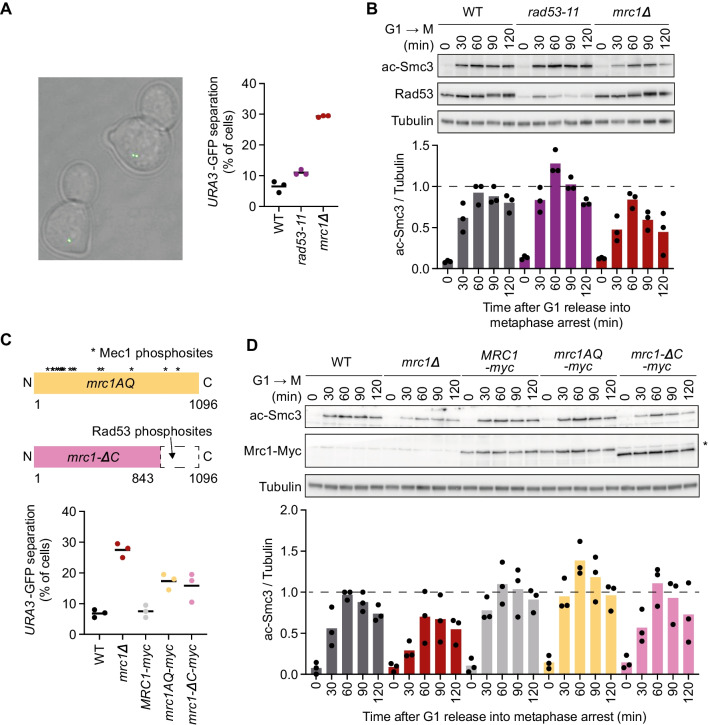
The replication checkpoint and cohesion establishment. **A** Representative example of cells imaged in the GFP dot assay. One GFP dot (bottom left cell) shows intact cohesion, while separated GFP dots (top right cell) indicate a cohesion defect. One hundred to 200 cells were scored for each indicated genotype. Three independent repeats of the experiment were performed. The means and the individual values are shown, revealing a significant cohesion defect in *mrc1Δ* cells (unpaired *t* test *p* < 0.0001) as well as a smaller yet significant defect in *rad53-11* cells (*p* = 0.018). **B** Immunoblotting of synchronized cells from **A** at the indicated time points to analyze Smc3 acetylation and Rad53. Tubulin served as a loading control. The ac-Smc3/tubulin ratios were normalized to the highest ratio observed in wild-type cells. Means and individual values from three independent repeat experiments are shown. Acetylation levels were significantly lower in *mrc1Δ* cells and higher in *rad53-11* (two-way ANOVA tests, *p* = 0.048 and *p* = 0.0081, respectively). **C** Schematic of the Mrc1 mutants analyzed, as well as result from the GFP dot assay to assess sister chromatid cohesion. Compared to increased GFP dot separation in *mrc1Δ* cells vs. wild type (unpaired *t* test, *p* = 0.0002), *mrc1AQ-myc* and *mrc1-DC-myc* cells showed a smaller but also significant cohesion defect (*p* = 0.0064 and *p* = 0.048, respectively). **D** Smc3 acetylation was assessed by immunoblotting; Mrc1 was detected via its myc epitope tag. Acetylation differences, assessed using a two-way ANVOA test, remained insignificant at *p* < 0.05. An antibody background band is marked with an asterisk

To determine whether the replication checkpoint contributes to cohesion establishment, we measured sister chromatid cohesion in *rad53-11* cells harboring a defective replication checkpoint effector kinase Rad53 (Shimada et al. 2002; Forey et al. 2020). Rad53-11 protein is sparsely expressed and fails to become phosphorylated in response to exposure to the replication inhibitor hydroxyurea (HU) (Fig. S1A). To assess the state of sister chromatid cohesion, we synchronized *rad53-11* cells in G1 using α-factor, followed by release to progression through S phase and arrest in mitosis using the spindle poison nocodazole. Cells expressed a tet repressor-GFP fusion protein that marks a tet operator array integrated at the *URA3* locus, close to the centromere of chromosome 5. Premature splitting of the resulting GFP dot in a mitotic arrest indicates defective sister chromatid cohesion (Fig. 1A). While cells lacking Mrc1 (*mrc1Δ*) displayed a substantial cohesion defect, compared to wild-type control cells, *rad53-11* cells showed a much smaller increase in GFP dot separation (Fig. 1A). As a complementary approach to assess successful sister chromatid cohesion establishment, we monitored Smc3 acetylation levels during S phase by immunoblotting. As previously seen (Borges et al. 2013), Smc3 acetylation was reduced in *mrc1Δ* cells compared to wild type, but instead appeared slightly elevated in *rad53-11* cells (Fig. 1B). These observations reveal largely successful sister chromatid cohesion establishment in the absence of a functional replication checkpoint.

The Rad53 checkpoint kinase, as well as the main upstream sensor of the replication checkpoint, Mec1, are essential proteins for their role in regulating dNTP synthesis. They can be deleted in the absence of a cellular inhibitor of dNTP synthesis, Sml1 (Zhao et al. 1998). *rad53Δ sml1Δ* and *mec1Δ sml1Δ* cells showed a small increase in the frequency of GFP dot separation when compared to wild-type cells, well below the cohesion defects seen in *mrc1Δ* cells (Fig. S1B).

As an additional way to probe a possible replication checkpoint contribution to sister chromatid cohesion, we utilized two checkpoint defective Mrc1 alleles (Fig. 1C). *mrc1AQ* lacks 17 SQ and TQ motifs that are recognized by the Mec1 upstream kinase to mediate the checkpoint signal (Osborn and Elledge 2003). *mrc1-ΔC* in turn is missing the Mrc1 C-terminus that harbors a cluster of Rad53 kinase recognition sites (McClure and Diffley 2021). Previous studies reported conflicting observations with *mrc1AQ* cells, reporting wild-type levels of sister chromatid cohesion (Xu et al. 2004) or cohesion defects similar to *mrc1Δ* cells (Tsai et al. 2015). We observed an intermediate frequency of GFP dot separation in both *mrc1AQ* and *mrc1-ΔC* cells, above wild type but below what is observed in *mrc1Δ* cells (Fig. 1C). Meanwhile, Smc3 acetylation in *mrc1AQ* and *mrc1-ΔC* cells remained at wild-type levels, unlike the acetylation defect seen in *mrc1Δ* cells (Fig. 1D). It is possible that small sister chromatid cohesion defects, revealed in the GFP dot assay, arose because the *mrc1AQ* and *mrc1-ΔC* alleles also compromise Mrc1 function outside of the replication checkpoint. While the exact relationship between the replication checkpoint and cohesion establishment merits further exploration, taken together, our results suggest that cohesion establishment is achieved largely independently of the replication checkpoint.

### Replisome speed and cohesion establishment

**Fig. 2:**
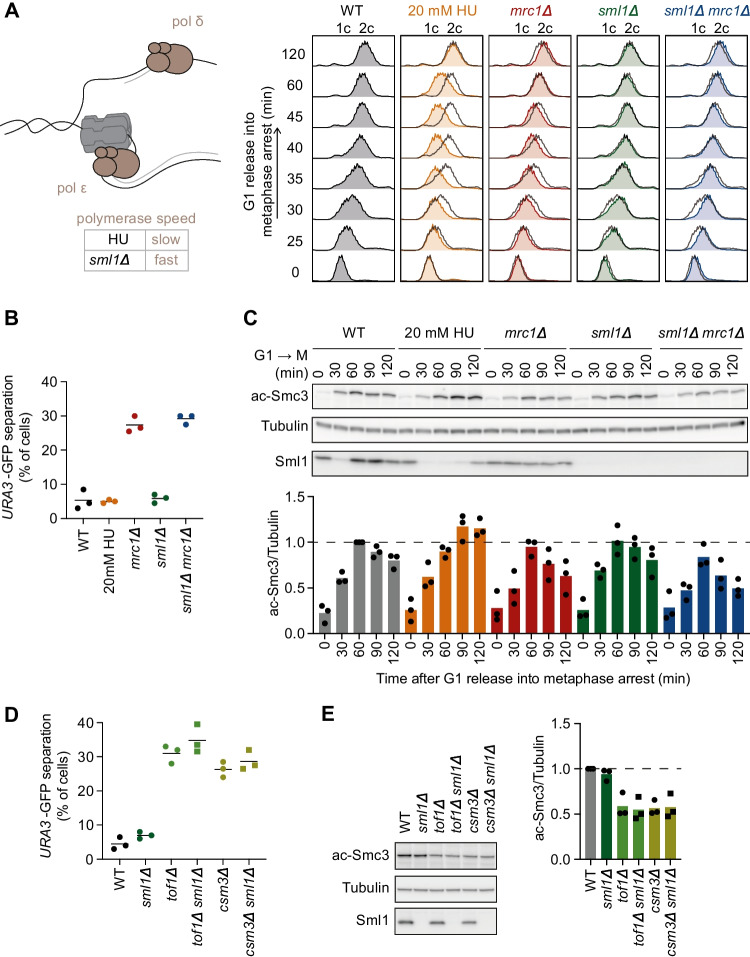
Replisome speed and cohesion establishment. **A** Schematic illustrating modulated DNA polymerase speeds. FACS analysis of DNA content of cells of the indicated genotypes following G1 release into medium containing nocodazole (and HU as indicated). The wild-type profile is overlaid onto the others as a dark grey outline. **B** Sister chromatid cohesion in the indicated strains, as judged by the GFP dot assay. Mean and individual values of three independent experiments are shown. Unpaired *t* tests revealed a significant cohesion defect in *mrc1Δ* cells, compared to wild type (*p* = 0.0005), but no significant defect following 20 mM HU treatment, and no significant difference between *mrcl1Δ* and *sml1Δ mrc1Δ* cells. **C** Immunoblotting at the indicated time points to analyze Smc3 acetylation and Sml1. Tubulin served as a loading control. The ac-Smc3/tubulin ratios were normalized to the highest ratio observed in wild-type cells. Means and individual values from three independent repeat experiments are shown. Two-way ANOVA tests revealed significantly increased Smc3 acetylation following 20 mM HU treatment (*p* = 0.029), while other effect sizes did not reach significance at *p* < 0.05. **D** As **B**, using strains of the indicated genotypes. Unpaired *t* tests confirmed significant cohesion defects in *tof1Δ* and *csm3Δ* cells, compared to wild type (*p* = 0.0001 and *p* = 0.0002, respectively), but no significant differences due to *sml1* deletion. **E** As **C**, but only samples taken at 120 min were analyzed. Unpaired *t* tests confirmed significant acetylation defects in *tof1Δ* and *csm3Δ* cells, compared to wild type (*p* = 0.0055 and *p* = 0.0007, respectively), but no significant differences due to *sml1* deletion

Mrc1 and Tof1/Csm3 ensure that the replisome progresses at full speed (Tourriere et al. 2005; Yeeles et al. 2017), raising the question of whether successful cohesion establishment requires replication fork encounters at a certain pace. Perhaps cohesion establishment reactions occur at a rate that must be coordinated with that of fork progression. We began to investigate this possibility by manipulating replisome speed independently of Tof1-Csm3 and Mrc1, by altering the dNTP pools available to DNA polymerases (Fig. 2A).

HU inhibits ribonucleotide reductase, the enzyme complex that synthesizes dNTPs. While high HU concentrations (100–200 mM) block replication progression, lower concentrations lead to gradual replisome slowdown. Wild-type cells supplemented with 20 mM HU show replication rates similar to those observed in *mrc1Δ* cells without HU (both ca. 1100 bp/min, compared to a wild-type replication speed of ca. 2100 bp/min) (Theulot et al. 2022). We therefore examined sister chromatid cohesion in wild-type cultures, supplemented with 20 mM HU during their progression through S phase. Flow cytometric analysis of DNA content at 5-min intervals confirmed slower DNA synthesis, comparable to that in *mrc1Δ* cells (Fig. 2A). At later times, *mrc1Δ* cells completed S phase more efficiently than wild-type cells exposed to 20 mM HU, which could be due to activation of late replication origins in *mrc1Δ* but not wild-type cells (Koren et al. 2010).

Unlike *mrc1Δ* cells, wild-type cells treated with 20 mM HU did not show increased GFP dot separation—they had therefore successfully established sister chromatid cohesion (Fig. 2B). Measuring Smc3 acetylation levels in cells treated with 20 mM HU revealed no reduction, compared to that seen in *mrc1Δ* cells (Fig. 2C). On the contrary, acetylation levels were slightly higher than in wild-type control cells, an observation that we will return to below. Together, these observations suggest that a slowdown of replication fork progression is not by itself a cause of sister chromatid cohesion defects.

As an alternative way to probe the relationship between fork speed and sister chromatid cohesion establishment, we aimed to restore fork progression in *mrc1Δ* cells to wild-type rates. Removing the ribonucleotide reductase inhibitor Sml1 results in augmented cellular dNTP pools and an increased fork progression rate (Poli et al. 2012; Theulot et al. 2022). Faster replication fork progression in *sml1Δ* single mutant cells, compared to wild type, did not cause sister chromatid cohesion defects (Fig. 2A–C), strengthening the impression that cohesion establishment occurs independently of a specific fork speed. Sml1 deletion in *mrc1Δ* cells returned DNA synthesis rates in the double mutant cells close to those observed in wild-type controls (Fig. 2A). However, sister chromatid cohesion or Smc3 acetylation did not improve (Fig. 2B, C). This suggests that the cohesion defect in *mrc1Δ* cells did not arise due to slow replication fork progression.

Tof1-Csm3 act in a cohesion establishment pathway parallel to Mrc1 (Xu et al. 2007). We therefore also explored the effect of restoring replication speed to *tof1Δ* and *csm3Δ* cells. However, neither sister chromatid cohesion nor Smc3 acetylation were improved in *tof1Δ* or *csm3Δ* cells by removing Sml1 (Fig. 2D, E). Replication speed in the absence of Tof1 or Csm3 decreases more modestly when compared to *mrc1Δ* cells, akin to what is observed in wild-type cells treated with 5–10 mM HU (Yeeles et al. 2017; Theulot et al. 2022). However, treating wild-type cells with 5 mM or 10 mM HU also did not result in noticeable sister chromatid cohesion or Smc3 acetylation defects (Fig. S2A). Together, we conclude that successful sister chromatid cohesion establishment is possible at a range of DNA polymerase speeds.

### HU augments Smc3 acetylation, independent of cohesion establishment

We had noticed that 20 mM HU treatment led to increased Smc3 acetylation in wild-type cells (Fig. 2C). Titrating HU concentrations between 5 and 20 mM (Fig. S2B) revealed a dose-dependent increase in Smc3 acetylation. While HU-mediated replication slowdown did not interfere with cohesion establishment, we wondered whether, on the contrary, increased Smc3 acetylation due to HU treatment could improve cohesion establishment. We therefore supplemented cultures of *tof1Δ*, *csm3Δ*, and *mrc1Δ* cells with 20 mM HU and monitored Smc3 acetylation and sister chromatid cohesion establishment. Indeed, HU exposure restored Smc3 acetylation in *tof1Δ*, *csm3Δ*, and *mrc1Δ* cells to levels seen in wild-type cells. However, increased Smc3 acetylation was not accompanied by improved sister chromatid cohesion (Fig. S2C). This reveals an Smc3 acetylation reaction, in response to HU treatment, that is independent of successful sister chromatid cohesion establishment. The nature of this reaction, as well as its relationship with Smc3 acetylation during undisturbed replication fork progression, remains to be investigated.

### DNA helicase speed and cohesion establishment

Above, we altered replisome progression by modulating DNA polymerase speed via cellular dNTP pools. In comparison, Tof1-Csm3 and Mrc1 associate with the CMG helicase, where they are thought to affect replisome progression by regulating the speed of DNA unwinding (Eickhoff et al. 2019; Baretić et al. 2020; McClure and Diffley 2021). The effects on replisomes of altering helicase or polymerase speed might differ. For example, slowing down polymerases while DNA unwinding continues at a constant rate might result in increased availability of single-stranded DNA (ssDNA). In contrast, slower DNA unwinding at a constant rate of DNA synthesis might reduce the amount of accessible ssDNA. As ssDNA is a substrate for cohesion establishment (Murayama et al. 2018), we sought a way to emulate the Tof1-Csm3 and Mrc1 effects more accurately by modulating the rate of DNA unwinding.

**Fig. 3:**
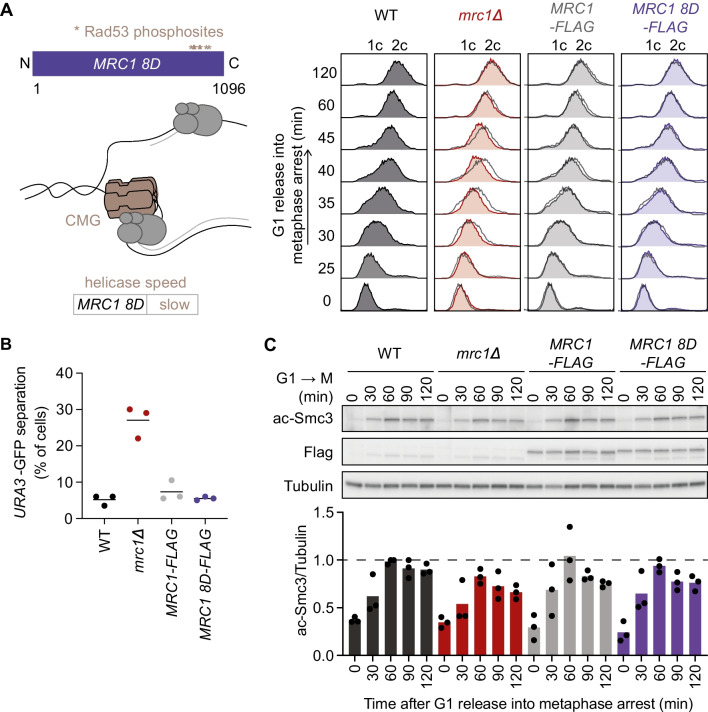
Helicase speed and cohesion establishment. **A** Schematic depicting the *MRC1 8D* allele and its effect on the speed of DNA unwinding. FACS analysis of DNA content as cells of the indicated genotypes were released from G1 block for synchronous progression through S phase into nocodazole-imposed mitotic arrest. The wild-type profile is overlaid onto the others as a dark grey outline. **B** Sister chromatid cohesion in the same experiment was assessed by the GFP dot assay at the 120-min time point. Means and individual values of three independent repeat experiments are shown. Unpaired *t* tests confirmed a significant cohesion defect in *mrc1Δ* cells compared to wild type (*p* = 0.0012), but no significant difference between *MRC1-FLAG* and *MRC1 8D-FLAG* cells. **C** Smc3 acetylation during the same experiment was analyzed by immunoblotting. Tubulin served as a loading control. The ac-Smc3/tubulin ratios were normalized to the highest ratio observed in wild-type cells. Means and individual values from three independent repeat experiments are shown. Two-way ANOVA tests showed that acetylation differences remained insignificant at *p* < 0.05

DNA unwinding is controlled by the replication checkpoint. Rad53 phosphorylates Mrc1 on a number of C-terminal phosphorylation sites to slow down replication fork progression in response to checkpoint activation (McClure and Diffley 2021) (Fig. 3A). An *MRC1-8D* allele in which 8 of these phosphorylation sites were changed to phosphorylation-mimicking aspartates imposes constitutive replication fork slowdown. We therefore analyzed the proficiency of *MRC1-8D* cells to establish sister chromatid cohesion. Despite slower DNA unwinding, we did not observe any cohesion establishment defect in *MRC1-8D* cells, as monitored by GFP dot separation and Smc3 acetylation (Fig. 3B, C). This suggests that sister chromatid cohesion establishment is proficient even at slower helicase progression rates.

As a complementary approach to address whether the availability of ssDNA is limiting the efficiency of sister chromatid cohesion establishment in *tof1Δ*, *csm3Δ*, and *mrc1Δ* cells, we utilized a mutation in the large subunit of the single-stranded DNA binding protein *rfa1(G77E)*, which shows reduced affinity for single-stranded DNA. This allele improved the cohesion defect seen in the absence of Ctf18-RFC, a cohesion establishment factor that loads PCNA to serve as a potential adaptor for cohesin loading (Murayama et al. 2018; Liu et al. 2020; Minamino et al. 2023). Unlike in the case of Ctf18-RFC, the cohesion defects in the absence of Tof1-Csm3 or Mrc1 remained unaltered by the *rfa1(G77E)* allele (Fig. S3A). This observation supports the idea that Tof1-Csm3 and Mrc1 act in sister chromatid cohesion establishment independently of affecting ssDNA accessibility.

As a final approach to modulating the rate of replication fork progression, we utilized a yeast strain lacking the catalytic domain of DNA polymerase ε (*polε-Δcat*). In these cells, polymerase delta takes over leading strand synthesis, albeit at markedly reduced DNA unwinding and synthesis rates (Kesti et al. 1999; Yeeles et al. 2017) (Fig. S3B). *polε-Δcat* cells showed a marked increase of GFP dot separation, as well as slightly reduced Smc3 acetylation. This result can be interpreted in two ways. It could be that sister chromatid cohesion establishment is compromised at very low replication speeds in the *polε-Δcat* strain, which are substantially lower than in *tof1Δ*, *csm3Δ*, or *mrc1Δ cells*. Alternatively, the cohesion defect in *polε-Δcat* cells could stem from a role of DNA polymerase ε that is independent of its role in DNA replication. In support of the latter possibility, a small C-terminal deletion in DNA polymerase ε elicits a cohesion defect without apparent effect on DNA replication (Edwards et al. 2003). The role of DNA polymerase ε in sister chromatid cohesion establishment merits further exploration.

### Tof1’s role in topoisomerase I and FACT recruitment

**Fig. 4:**
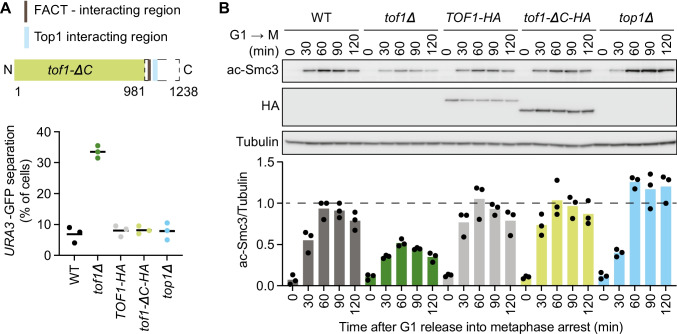
Tof1 and auxiliary replisome components. **A** Schematic depicting the Tof1 topoisomerase I and FACT interaction motifs, as well as the *tof1-ΔC* truncation. Sister chromatid cohesion was assessed in cells of the indicated genotypes 120 min after release from G1 block into nocodazole-imposed mitotic arrest. Means and individual values of three independent repeat experiments are shown. Unpaired *t* tests confirmed a significant cohesion defect in *tof1Δ* cells compared to wild type (*p* = 0.0001), but no significant difference between *TOF1-HA* and *tof1-ΔC-HA* cells, or between *top1Δ* and the wild-type control. **B** Immunoblotting of samples from the same experiment at the indicated times to analyze Smc3 acetylation and Tof1. Tubulin served as a loading control. The ac-Smc3/tubulin ratios were normalized to the highest ratio observed in wild-type cells. Means and individual values from three independent repeat experiments are shown. Two-way ANOVA tests confirmed a significant Smc3 acetylation defect in *tof1Δ* cells compared to wild type (*p* = 0.0001), but no significant difference between *TOF1-HA* and *tof1-ΔC-HA* cells. Acetylation was significantly increased in *top1Δ* cells (*p* = 0.0021)

In addition to impacting on DNA replication, Tof1 is known to recruit auxiliary proteins to the replisome. A predicted unstructured C-terminal Tof1 extension contains the interaction sites for both topoisomerase I and FACT (Shyian et al. 2020; Westhorpe et al. 2020; Safaric et al. 2022) (Fig. 4A). It is conceivable that topological stress at replication forks, which accumulates in the absence of topoisomerase I, impairs cohesion establishment. Alternatively, histone clearance and redeposition, facilitated by FACT, might have to be coordinated with sister chromatid cohesion establishment.

To determine whether topoisomerase I and/or FACT recruitment to the replisome play a role in cohesion establishment, we made use of a C-terminal Tof1 truncation mutant (*tof1-ΔC*, lacking residues 982–1238) that no longer recruits topoisomerase I and FACT (Westhorpe et al. 2020; Safaric et al. 2022). We then assessed sister chromatid cohesion and Smc3 acetylation. The frequency of GFP dot separation, as well as Smc3 acetylation levels, was indistinguishable between wild-type and *tof1-ΔC* cells, while cells lacking Tof1 altogether (*tof1Δ*) displayed the expected cohesion defect signature (Fig. 4A, B). These observations suggest that topoisomerase I or FACT recruitment to the replisome is dispensable for successful sister chromatid cohesion establishment.

Topoisomerase I is a non-essential protein in budding yeast, as topoisomerase II can compensate for its fundamental role in relieving topological stress. We were therefore able to use a strain devoid of topoisomerase I (*top1Δ*) to independently address whether this enzyme contributes to cohesion establishment. We found that topoisomerase I is dispensable for cohesion establishment, as seen in the GFP dot assay (Fig. 4A). We noticed somewhat elevated Smc3 acetylation levels in *top1Δ* cells, when compared to wild-type cells (Fig. 4B). The reason for this increase, like that previously seen in HU-treated cells, remains to be elucidated. In summary, we conclude that successful cohesion establishment is possible without interactions that the Tof1 C-terminus provides with auxiliary replication factors, including topoisomerase I.

### Direct protein interactions between Tof1-Csm3, Mrc1, and cohesin

The known roles of Tof1-Csm3 and Mrc1 at the replisome, which we so far studied, appear unrelated to the establishment of sister chromatid cohesion. We therefore hypothesized that Tof1-Csm3 and Mrc1 perform a previously unknown function in cohesion establishment. Recent structural studies have placed Tof1-Csm3 and the Mrc1 N-terminus at the front of the replisome (Eickhoff et al. 2019; Baretić et al. 2020), a prime location where cohesion establishment factors would physically encounter cohesin as the replication fork approaches. We therefore investigated whether Tof1-Csm3 and Mrc1 engage in protein interactions with cohesin.

Previous studies using nematode and human cell extracts have reported an interaction between their respective Tof1 orthologs, TIM-1 and TIMELESS, and cohesin (Chan et al. 2003; Leman et al. 2010). However, a mass spectrometry-based interaction screen using human cell extracts failed to confirm a TIMELESS-cohesin interaction (Ivanov et al. 2018). To investigate the possibility of direct Tof1-Csm3 or Mrc1 interactions with cohesin, we biochemically purified budding yeast Tof1-Csm3, Mrc1, and cohesin (Yeeles et al. 2017; Minamino et al. 2018). We also included the cohesion establishment factor Chl1, which was reported to interact with cohesin in human and yeast cell extracts (Parish et al. 2006; Samora et al. 2016).

**Fig. 5:**
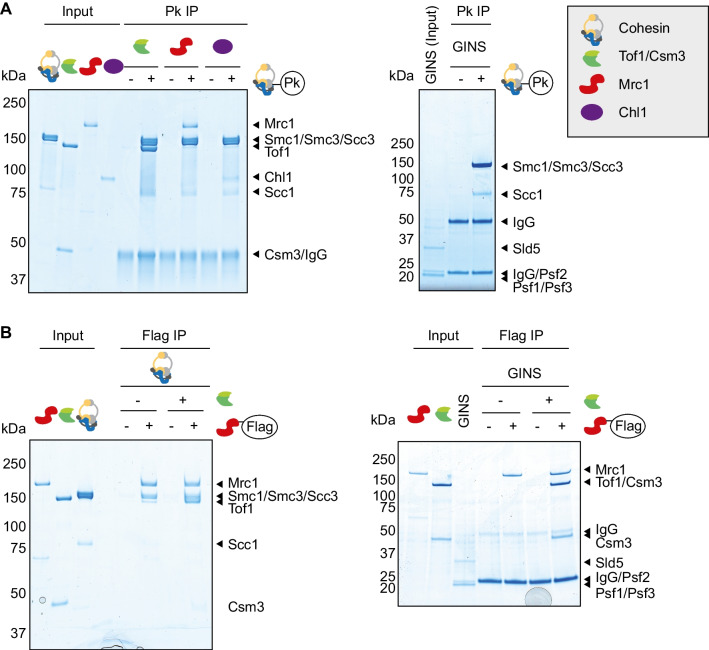
Protein interactions between cohesion establishment factors and cohesin. **A** Interaction screen with cohesin as the bait. Cohesin-coated or control beads were incubated with the indicated replisome components. Ten percent of input proteins are loaded next to the bead-bound fractions. Proteins were visualized by Coomassie Blue staining. **B** Interaction screen with Mrc1 as the bait. Mrc1-coated or control beads were first incubated with or without Tof1-Csm3, before a further incubation with cohesin or the GINS complex and analyzed as above

To investigate the possibility of direct protein interactions, we employed an experimental setup in which a purified target protein is immobilized on beads, before these are briefly immersed with candidate binding partners (C. Smith and J. Diffley, personal communication). We immobilized cohesin using an antibody against a Pk epitope tag fused to its Smc1 subunit. These cohesin-covered beads, or antibody-only control beads, were incubated with Tof1-Csm3, Mrc1, or Chl1. Not reported to interact with cohesin, the GINS complex was used as a control. Beads were then washed, bound protein eluted, and analyzed by SDS-polyacrylamide gel electrophoresis followed by Coomassie Blue staining. Tof1-Csm3, Mrc1, as well as Chl1 were all recovered in the bead-bound fraction in a cohesin-dependent manner (Fig. 5A), suggesting that these cohesion establishment factors directly interact with the cohesin complex. The GINS complex, on the other hand, did not interact with cohesin.

To confirm the interaction of Tof1-Csm3 and Mrc1 with cohesin, we took a reverse approach and immobilized Mrc1 on beads using an antibody against a Flag epitope tag on Mrc1. Following incubation with cohesin and washes, the bound protein was eluted by incubation with Flag peptide. Mrc1 on beads, but not control beads, interacted with cohesin (Fig. 5B). Addition of both Tof1-Csm3 and cohesin to Mrc1 beads led to the retention of both complexes, consistent with direct interactions among the three components. As a control, GINS was not retained on Mrc1 beads (to detect any possible GINS traces, elution in this case was with SDS).

We also immobilized Tof1-Csm3 on beads, which specifically interacted with cohesin (Fig. S4A). This interaction was unaltered by the inclusion of benzonase during the incubations, excluding the possibility that the interaction was mediated through DNA that might have contaminated our purified proteins.

So far, we performed our interaction incubations in buffer containing 100 mM potassium glutamate, conditions close to a budding yeast physiological environment. To test how stably Tof1-Csm3 interacts with cohesin, we added increasing NaCl concentrations during the binding incubation. The interaction was gradually lost in the presence of more than 250 mM NaCl (Fig. S4B), suggesting that polar or charge interactions contribute. We also performed benzonase treatment and salt titration for the Mrc1-cohesin interaction with similar outcomes (Fig. S4C,D).

Finally, we tested whether complexes formed between Tof1-Csm3, Mrc1 and cohesin are stable enough to be characterized by size exclusion chromatography. Following incubation, individual or combined protein samples were separated on a gel filtration column. Immunoblot analysis showed that cohesin and Tof1-Csm3 (visualized by a CBP tag on Csm3) individually eluted at volumes expected of their respective sizes (Fig. S5). After co-incubation, a faint Csm3 band became detectable in earlier fractions where cohesin elutes, while the bulk of the Csm3 profile remained unchanged, suggesting that the Tof1-Csm3 interaction with cohesin is weak. In a similar experiment, Mrc1 mostly co-eluted with cohesin, suggesting that Mrc1 and cohesin engage in a more durable interaction.

### Tof1-Csm3 and Mrc1 deploy multipronged cohesin interactions

**Fig. 6:**
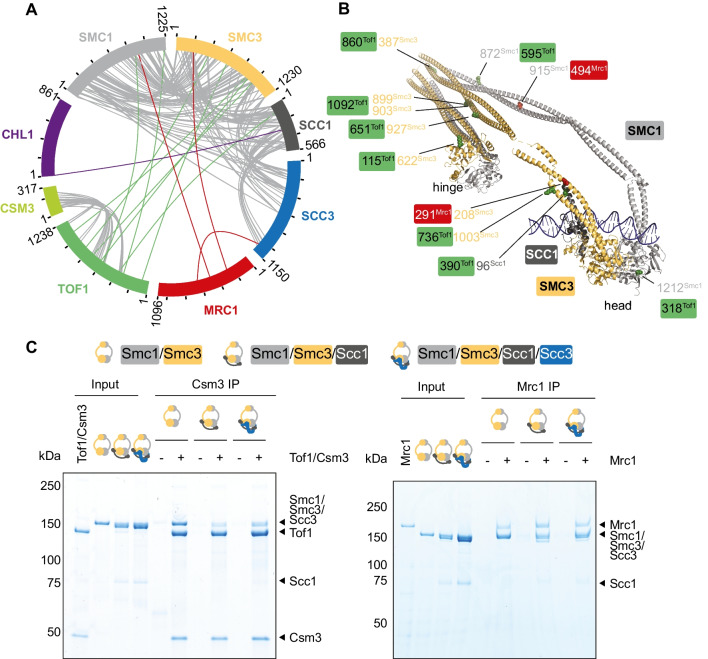
Multipronged cohesion establishment factor interactions with the cohesin complex. **A** Collated circos plot of interprotein crosslinks detected by CLMS in the two samples. Grey lines represent intersubunit crosslinks within the cohesin or Tof1-Csm3 complexes. Linkages between cohesin and Tof1 (green), Mrc1 (red), and Chl1 (purple) are highlighted. **B** Atomic model of the budding yeast Smc1-Smc3 dimer and Scc1 N-terminus, built using Phyre^2^ (Kelley et al. 2015) based on a fission yeast cohesin model (Higashi et al. 2020). CLMS linkage sites are highlighted. **C** Interaction screen with Tof1-Csm3 (left) or Mrc1 (right) on beads, comparing cohesin tetramer, cohesin trimer, and Smc1-Smc3 dimer as binding partners. Ten percent of the input and the bead bound fractions were analyzed by SDS-polyacrylamide gel electrophoresis followed by Coomassie Blue staining

To explore the cohesion establishment factor interactions with cohesin, we performed protein crosslinking mass spectrometry (CLMS) to identify interaction sites. In two repeats of the experiment, we included cohesin, Tof1-Csm3 and Chl1, either with or without Mrc1. Cα atom distances spanned by crosslinks inside known structured regions of individual proteins fell within the expected range of the sulfo-SDA crosslinker (Fig. S6). Furthermore, we identified numerous crosslinks between subunits of the cohesin complex, as well as between Tof1 and Csm3, that were consistent with prior structural knowledge (Fig. 6A) (Baretić et al. 2020; Higashi et al. 2020), thereby overall validating the CLMS experiment.

Additionally, we detected crosslinks between the cohesion establishment factors and cohesin (Fig. 6A). Most prevalent were interactions of Tof1 and Mrc1 with Smc1 and Smc3, with an additional link each between Mrc1 and Scc3, as well as Chl1 and Scc1. Figure 6B shows these interactions mapped onto a Smc1-Smc3 structural model, revealing interaction clusters along both SMC coiled coils. To experimentally validate the conclusion that Tof1-Csm3 and Mrc1 preferentially interact with the Smc1-Smc3 dimer within the cohesin complex, we extended our biochemical interaction analysis. We immobilized Tof1-Csm3 or Mrc1 on beads that we then incubated with either purified cohesin tetramer complexes (Smc1-Smc3-Scc1-Scc3), cohesin trimers (Smc1-Smc3-Scc1), or SMC dimers (Smc1-Smc3). The SMC dimer interacted with both Tof1-Csm3 and Mrc1 equally, if not more efficiently, when compared to the cohesin trimer or tetramer complexes (Fig. 6C). This finding is consistent with the notion that Tof1-Csm3 and Mrc1 make prominent contacts with the Smc1-Smc3 dimer.

**Fig. 7:**
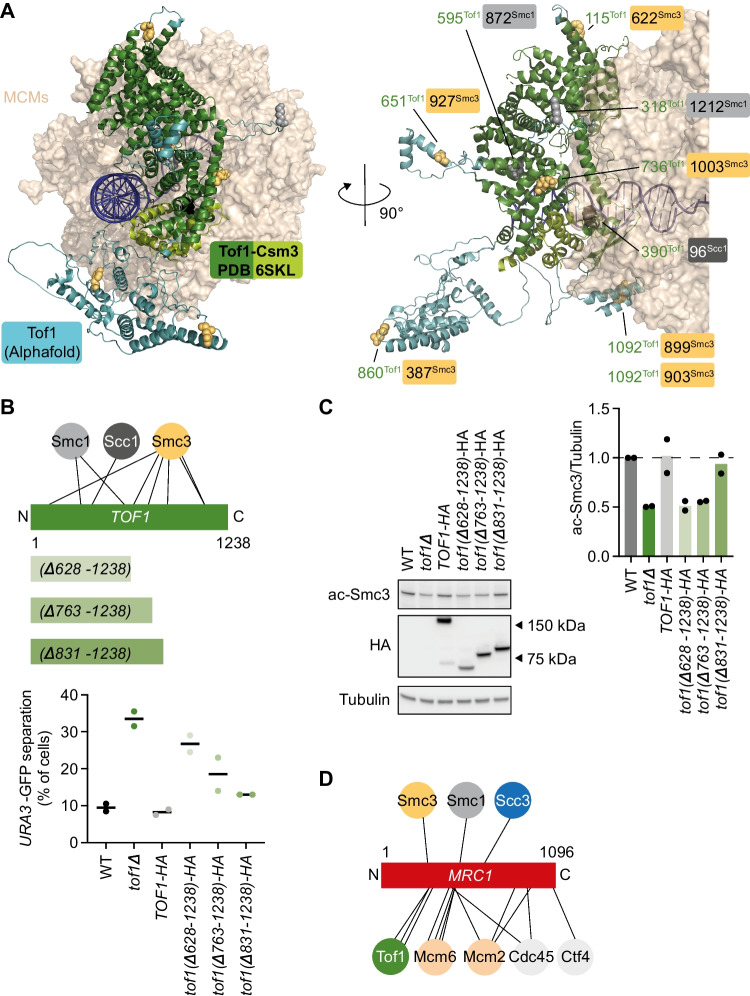
Tof1 regions important for sister chromatid cohesion. **A** CLMS contact sites, highlighted on the Tof1-Csm3 structure (green) (Baretić et al. 2020), with unresolved Tof1 regions (amino acids 107–115, 305–328, 605–659, and 782–1000) filled with their AlphaFold predictions (cyan, manually adjusted to avoid a DNA clash). Linkages with Smc1, Smc3, and Scc1 are highlighted in grey, yellow, and black, respectively. DNA and the MCM hexamer are shown for context. **B** Schematic representation of Tof1 and its cohesin crosslinks, as well as of three Tof1 truncation mutants. Sister chromatid cohesion in strains of the indicated genotypes, including the three truncation mutants, was assessed by the GFP dot assay. Means and individual values from two independent repeat experiments are shown. **C** Smc3 acetylation and Tof1 were analyzed by immunoblotting in the same experiment. Tubulin served as a loading control. The ac-Smc3/tubulin ratios were normalized to the highest ratio observed in wild-type cells. Means and individual values from two independent repeat experiments are shown. **D** Schematic representation of Mrc1 and its cohesin crosslinks, as well as previously reported crosslinks with replisome components (Baretić et al. 2020)

On the cohesion establishment factor side, Tof1 yielded the greatest number of cohesin crosslinks, which we mapped onto its available cryo-EM structure (Fig. 7A) (Baretić et al. 2020). Five of the eight identified crosslinks emanated from unresolved regions that we filled using AlphaFold (Jumper et al. 2021) predictions, suggesting a role for these probably more flexible Tof1 regions in providing cohesin contacts. Though we note that flexible protein regions might also show a greater propensity for spurious CLMS contacts. To investigate the importance of these interaction sites, we performed a Tof1 truncation analysis. We used previously characterized Tof1 C-terminal truncations (Westhorpe et al. 2020) and measured sister chromatid cohesion in these mutants. We had earlier tested a C-terminal deletion ending at amino acid 981, which is defective in topoisomerase I recruitment but was proficient in supporting cohesion establishment. When Tof1 was further truncated, we started to observe cohesion defects (Fig. 7B, C). A central Tof1 domain delineated by this analysis (covering amino acids 628–831) is a focal point of four of the identified CLMS cohesin contact sites, consistent with the idea that Tof1 contacts cohesin during cohesion establishment. Additionally, the central Tof1 domain might be required for Tof1-replisome interactions.

Inspecting the cohesin crosslinks with Mrc1 revealed an Smc3 interaction close to where Mrc1 interacts with Tof1 (Fig. 7D) (Baretić et al. 2020). The Mrc1 interactions on Tof1 in turn lie close to Tof1-Smc3 linkages (Fig. 7B), thus pinpointing a candidate Smc3-Tof1-Mrc1 interaction hub. Two other cohesin linkages fall into an Mrc1 region between its reported MCM and Cdc45 contact points. Mrc1 likely comprises of long unstructured extensions between its replisome contacts. Interactions with one of these long loops may provide a flexible cohesin tether that supports cohesion establishment. It will be of interest to define the interaction sites between Tof1-Csm3, Mrc1, and cohesin in more detail in a future study, which will allow interrogating the contributions of these interactions to sister chromatid cohesion establishment using site-specific mutations.

### A protein interaction between Tof1 and Chl1

Cohesion establishment factors at the replication fork engage in physical contacts with each other. Tof1-Csm3 and Mrc1 form a complex, while Ctf4 interacts with Chl1 (Bando et al. 2009; Samora et al. 2016). The human Tof1 and Chl1 orthologs, TIMELESS and DDX11, have also been reported to interact (Cortone et al. 2018). To address whether budding yeast Chl1 and Tof1 interact, we immobilized Chl1 on beads that we then incubated with Tof1-Csm3. Tof1-Csm3 was efficiently retained on Chl1-containing beads, but not on control beads (Fig. S7). Therefore, an interaction between Chl1 and Tof1-Csm3 is a feature conserved from yeast to human.

## Discussion

The roles of Tof1-Csm3 and Mrc1 in sister chromatid cohesion establishment have been known for almost two decades, but the basis for their contribution has remained unknown. In our study, we have systematically ruled out many of the multiple roles by which Tof1-Csm3 and Mrc1 participate in DNA replication. Instead, our findings open the possibility that a series of direct physical interactions between these replication fork components and the cohesin complex facilitate successful sister chromatid cohesion establishment during DNA replication.

### Replisome interactions with the cohesin complex

Recent structural insight shows how Tof1 and Csm3 form a complex at the head of the replisome, preceding the point where the helicase begins unwinding the DNA double helix (Eickhoff et al. 2019; Baretić et al. 2020). This places Tof1-Csm3 at a vantage point to engage with DNA binding proteins as the replication fork approaches. Chl1 is recruited by Ctf4 to a position that likely also points towards the front of the replisome (Samora et al. 2016). Thus, it could be that the cohesion establishment factors Tof1-Csm3 and Chl1, which form one epistasis group for their shared contributions to sister chromatid cohesion establishment (Xu et al. 2007), are jointly the first ones to engage with cohesin as the replication fork approaches. Mrc1 in turn makes a parallel contribution to cohesion establishment. This contribution could lie in securing additional cohesin contacts as the replisome progresses, resulting in a choreographed series of cohesin interactions that lead to cohesion establishment.

Might replisome components other than those discussed above engage with cohesin during sister chromatid cohesion establishment? The presently known “cohesion establishment factors” are typically non-essential proteins, as systematic genetic screens for chromosome stability mutants, in which many cohesion establishment factors were discovered, were restricted to non-essential genes (Mayer et al. 2004; Warren et al. 2004). Other screens uncovered the essential components of the cohesion machinery (Guacci et al. 1997; Michaelis et al. 1997; Tóth et al. 1999). However, the latter screens would not have uncovered essential replication fork components, as no cohesion defects can be measured if there is no DNA replication. It would be of interest to expand our cohesin interaction screen to include all of the replisome, consisting of well over 30 proteins and protein complexes. One candidate interactor is DNA polymerase *ε*, which has been implicated in sister chromatid cohesion (Edwards et al. 2003).

### Replisome-cohesin contacts during cohesion establishment

**Fig. 8:**
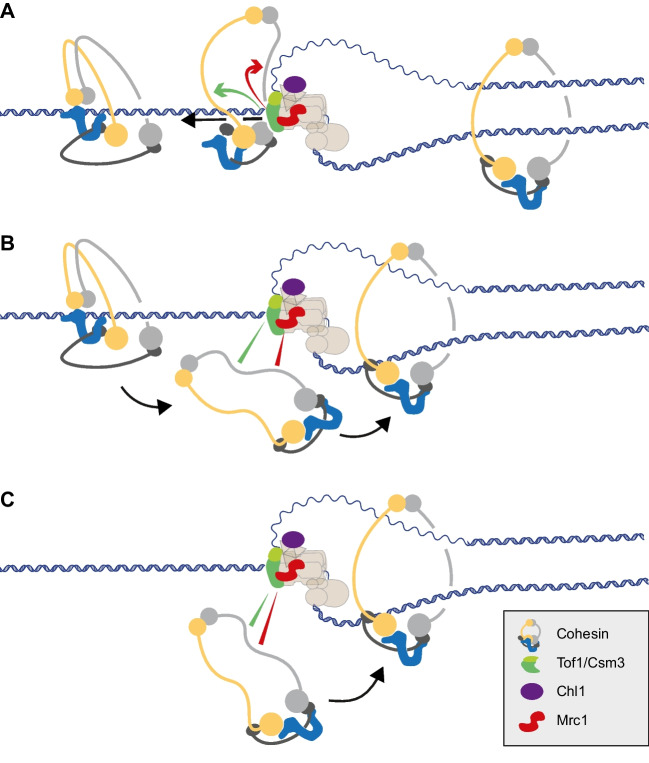
Models of how Tof1-Csm3 and Mrc1 might support sister chromatid cohesion establishment. **A** Tof1-Csm3 and Mrc1 help open a folded cohesin conformation to facilitate fork passage through the cohesin ring. **B** They tether cohesin while it is transferred behind the replication fork. **C** They provide protein interactions that recruit cohesin to the replication fork for *de novo* loading onto nascent leading and lagging strands

Our results suggest that Tof1 and Mrc1 make cohesin contacts along the Smc1 and Smc3 coiled coils. How might such contacts contribute to cohesion establishment? We consider here the possible implications of these contacts in the context of current models for cohesion establishment. Structural and microscopic studies show cohesin often adopting a folded conformation where the Smc1 and Smc3 coiled coils bend at their elbow (Sakai et al. 2003; Higashi et al. 2020). We do not currently know what structure cohesin adopts when bound to chromosomes. If cohesin retains a folded conformation, transitioning to an open ring-like conformation would be necessary if the replisome were to pass through cohesin rings (Fig. 8A and Fig. S8). In this scenario, Tof1 and Mrc1 might facilitate the unraveling of the coiled coils by contacting Smc1 and Smc3.

Alternatively, if cohesin is unloaded as the fork approaches and then reloaded behind the replisome, our cohesion establishment factors might be assisting in these unloading and/or reloading reactions. Interestingly, three Tof1 and Mrc1 contact points lie near the cohesin N-gate, through which DNA might pass (Fig. S8, cluster i). Even without active involvement in unloading or reloading, cohesion establishment factors may transiently tether cohesin while it is being transferred from the front to the back of the replisome (Fig. 8B). Analogous to the related case of histone inheritance during DNA replication (Willhoft and Costa 2021), cohesion establishment factors might act as a conveyor belt that transfers cohesin along a moving replisome.

In a third model of cohesion establishment, cohesin is de novo recruited to the replisome during S phase and sequentially loaded onto leading and lagging strands. Interactions with replisome components may in this case attract cohesin to the replication fork (Fig. 8C). The three models are not mutually exclusive in explaining sister chromatid cohesion establishment.

### Further roles of Tof1-Csm3 and Mrc1

Tof1-Csm3 and Mrc1 fulfil additional roles at the replication fork that we have not considered in our current study. A recently described function lies in Mcm7 ubiquitination, which promotes replisome disassembly during replication termination. This role diverged among eukaryotes. Tof1 and Csm3 orthologs in *C. elegans*, TIM-1 and TIPIN-1, are necessary for Mcm7 ubiquitination, while Mrc1 and Ctf4 orthologs, CLSP-1 and CTF-4, are not (Xia et al. 2021). Conversely, budding yeast Mrc1 and Ctf4 are required for Mcm7 ubiquitination, while Tof1 and Csm3 are not (Deegan et al. 2020). As all these factors participate in cohesion establishment, but only subsets in Mcm7 ubiquitination, replisome disassembly may not be their main function during sister chromatid cohesion establishment.

### Smc3 acetylation during sister chromatid cohesion establishment

Smc3 acetylation by the Eco1 acetyltransferase has been tied to DNA replication (Moldovan et al. 2006; Minamino et al. 2023), but how sister chromatid co-entrapment and Smc3 acetylation are coordinated remains unknown. We encountered examples where sister chromatid cohesion was defective, despite efficient Smc3 acetylation. This was the case in *tof1Δ*, *csm3Δ*, and *mrc1Δ* cells passing through S phase in the presence of HU. It appears that acetylation in this case targeted a cohesin pool that does not hold sister chromatids together. In another example, Smc3 acetylation levels in *eco1-1* mutant cells at permissive temperature are lower than levels observed in *ctf4Δ* and *chl1Δ* cells, despite a similar resulting strength of sister chromatid cohesion (Borges et al. 2013). Not all acetylated cohesin in *ctf4Δ* and *chl1Δ* cells is therefore engaged in sister chromatid cohesion. We imagine that challenged replication forks recruit and acetylate cohesin in support of fork protection (Delamarre et al. 2020), independently of sister chromatid cohesion. We also note that Smc3 acetylation defects in *mrc1Δ* cells often seemed less pronounced than the accompanying cohesion defects, maybe for this reason. Our observations emphasize the importance of further investigations into Smc3 acetylation and how cohesion establishment factors contribute to this reaction.

## Materials and methods

### Yeast strains

All budding yeast strains used in this study were of W303 background. Gene deletions and epitope tagging were performed by gene targeting using polymerase chain reaction (PCR) products. For strains with C-terminal truncations of Tof1 and Mrc1, one-step truncation and epitope tagging at the C-terminus were combined through gene targeting using PCR products. To create p*olε-Δcat* (*pol2 Δ1-1262*) strains (Kesti et al. 1999; Devbhandari and Remus 2020), In-Fusion cloning (TaKaRa Bio) was used to add a 371 bp *POL2* promoter region in front of a *POL2* fragment encompassing its C-terminal portion (amino acids 1263–1797) in the yeast shuttle vector YIplac204, followed by a further 226 bp of *POL2* upstream region. The plasmid was linearized with ApaI, between the end of *POL2* and the upstream region for gene replacement at the *POL2* locus. The *rad53-11* allele was introduced into a strain harboring a GFP-marked *URA3* locus by crossing strain PP37 (Forey et al. 2020) with our strain Y194, followed by tetrad dissection. The *mrc1AQ-*myc9 allele was constructed based on plasmid pD2043 (McClure and Diffley 2021) adding a myc9 epitope at the *MRC1* gene C-terminus. The resulting plasmid (p1942) was linearized with BsrGI to integrate at the *LEU2* locus in an *mrc1Δ* background strain. The *MRC1 8D-Flag* strain was constructed by linearizing pD3093 (McClure and Diffley 2021) with XbaI to target the *MRC1* locus for gene replacement. Colonies were genotyped using PCR for gene deletions. Epitope tagging was confirmed using immunoblotting against the respective epitope tags. In case of gene alterations, the genotypes of the final strains were confirmed by PCR followed by Sanger sequencing. All strains used in this study and their genotypes are listed in Table S1. A list of plasmids used in this study and their descriptions is contained in Table S2.

### Yeast culture

All cultures were grown in liquid YPD medium at 25 °C in shaking incubators at 180 rpm unless otherwise stated. Cells of **a** mating type were grown overnight in YPD medium and back-diluted in the morning. Four microgram per milliliter α-factor was added at OD_600_ = 0.2 and subsequently twice more every 55 min to arrest cells in G1. After 2 h 45 min, cells were released from G1 arrest by filtration and washing with 10× culture volume of YP. Cells were then resuspended in YPD + 6 μg/mL nocodazole to allow them to progress through S phase followed by arrest in metaphase. Samples for FACS analysis, for immunoblotting, and for the GFP dot assay were taken at the indicated timepoints after release from G1 arrest. Alternatively, cells were released from G1 arrest into YPD + 6 μg/mL nocodazole + hydroxyurea at the indicated concentrations.

### Flow cytometry analysis (FACS)

One-milliliter culture (OD_600_ 0.2–0.7) was harvested by centrifugation and resuspended and fixed overnight in cold 70% ethanol. Then, RNA was digested with 0.1 mg/mL RNaseA for 4 h (or overnight) at 37 °C in 50 mM Tris-HCl pH 7.5 buffer. To stain the DNA, the cells were resuspended in 200 mM Tris-HCl pH 7.5, 210 mM NaCl, 78 mM MgCl_2_ buffer containing 0.025 mg/mL propidium iodide. Cells were sonicated and diluted in 50 mM Tris-HCl pH 7.5 before analysis using a FACSCalibur cell analyzer (BD Biosciences). For each sample, 10,000 cells were counted. Flow cytometry data was analyzed using FlowJo (v10) software.

### Immunoblotting

Aliquots of the culture were harvested at the indicated timepoints during synchronized cell cycle progression and fixed using cold 20% TCA for at least 1 h. Cells were resuspended in sample buffer containing 100 mM Tris-HCl pH 6.8, 4% SDS, 20% glycerol, and 4% β-mercaptoethanol. Bead beating was used to break cells, before cell debris removal by centrifugation. Protein concentrations in the extract were determined using the Bradford assay. Fifteen microgram protein equivalent of each sample was separated by SDS-polyacrylamide gel electrophoresis, and gels were transferred to nitrocellulose membranes. Details of the antibodies used for immunoblotting can be found in Table S3. ECL or ECL Prime reagents (Cytiva) were used to visualize signals using an Amersham Imager 600 (Cytiva) or ChemiDoc MP Imaging System (Bio-Rad). Ac-Smc3 signals were quantified using FIJI software and normalized against tubulin that served as the loading control.

### GFP dot assay

Strains harboring tetO repeats at the *URA3* locus (at 35-kb distance from the centromere of chromosome V) and expressing the tetR-GFP fusion protein were harvested in metaphase, 2 h after G1 release into nocodazole-containing medium. Two-milliliter culture was harvested by centrifugation and resuspended and fixed in 70% cold ethanol overnight. For imaging, thin 2% agarose patches were prepared on glass slides. Cells were sonicated and resuspended in phosphate-buffered saline, and a drop was applied to the agarose patches. *z*-stacks with 35 images at 0.2-μm intervals were acquired using a DeltaVision Olympus IX70 inverted microscope with a 100× (NA = 1.40) PlanApo objective, deconvolved, and merged using maximum intensity projection. The number of GFP dots in each cell were then manually counted. One hundred to 200 cells were scored for each sample in each experiment.

### Protein purification

The budding yeast cohesin tetramers Tof1-Csm3 and Mrc1 were purified as previously described (Yeeles et al. 2017; Minamino et al. 2018). Cohesin trimer and the Smc1-Smc3 dimer were purified following the same protocol as cohesin tetramers. While a Protein A tag is fused to Scc1 for the purification of the cohesin trimer and tetramers, a corresponding Protein A tag is contained at the Smc1 C-terminus for purification of the Smc1-Smc3 dimer. In each case, after adsorption to IgG agarose beads during the first step of purification, the Protein A tag is removed by 3C protease cleavage.

To purify Chl1, an ectopic copy of codon-optimized Chl1 was overexpressed under control of the *GAL1* promoter in budding yeast. Strain Y5562 was grown in YP + 2% raffinose at 30 °C to an OD_600_ = 1.0 in a 100 L fermenter. Two percent galactose was added to induce protein expression for 2 h before harvesting. Cells were washed with deionized water and resuspended in lysis buffer (50 mM Tris-HCl pH 7.0, 500 mM NaCl, 10% glycerol, 2 mM MgCl_2_, 0.1% Triton X-100, 0.5 mM TCEP, 0.5 mM Pefablock + cOmplete^™^ protease inhibitors (Roche)). The suspension was frozen dropwise in liquid nitrogen and then pulverized in a freezer mill. Cell powder was thawed in lysis buffer on ice. Cell debris was removed by centrifugation in a Ti45 rotor at 40,000 rpm for 1 h. The supernatant was supplemented with benzonase and RNaseA and transferred onto IgG agarose beads for incubation on a rotating wheel at 4 °C for 2 h. The beads were then washed and incubated overnight with 3C protease in the same buffer. The eluate was diluted to 160 mM NaCl and loaded onto a heparin column equilibrated with 50 mM Tris-HCl pH 7.0, 160 mM NaCl, 10% glycerol, 2 mM MgCl_2_, and 0.5 mM TCEP. Protein was eluted with a salt gradient from 160 mM to 1 M NaCl. Chl1-containing fractions were pooled, concentrated using a 30-kDa cutoff Vivaspin concentrator, and further purified using a Superdex 200 Increase column equilibrated in 20 mM Tris pH 7.5, 150 mM NaCl, 10% glycerol, and 0.5 mM TCEP. The final Chl1 preparation was again concentrated by ultrafiltration.

### Protein interaction analyses

*Cohesin as bait.* For each interaction test, 5 μL Protein A dynabeads were equilibrated with interaction buffer (25 mM HEPES-KOH pH 7.6, 100 mM potassium glutamate, 10 mM magnesium acetate, 0.12% NP-40). α-Pk antibody was added in interaction buffer on a wheel at 4 °C for at least 2 h. The beads were washed; then, 200–600 nM purified cohesin tetramer was added and adsorbed for 10 min on a thermomixer at 30 °C and 1000 rpm. The beads were washed again before 200 nM prey proteins were added in interaction buffer at 30 °C for 5 min. The beads were washed again, and bead-bound proteins eluted with sample buffer (100 mM Tris-HCl pH 6.8, 4% SDS, 20% glycerol, and 4% β-mercaptoethanol) at 95 °C for 5 min, before analysis by SDS polyacrylamide gel electrophoresis, alongside 10% of input proteins. Input and recovered proteins were visualized by Coomassie Brilliant Blue staining.

*Tof1-Csm3 as bait.* The interaction analysis was carried out as above, but 3 μL of Calmodulin dynabeads were used, to which 200–600 nM Tof1/Csm3 were adsorbed.

*Mrc1 or Chl1 as bait.* Four microliter α-Flag M2 magnetic beads were used, to which 200–600 nM Flag-tagged Mrc1 or Chl1 was adsorbed. When M2 beads with Mrc1 as bait were first incubated with Tof1-Csm3 (prey 1), the beads were washed before incubation with cohesin or GINS (prey 2). As alternative to sample buffer, bead-bound protein was eluted by competition using 0.5 mg/mL 3× Flag peptide in interaction buffer. As indicated, benzonase, or increasing concentrations of NaCl, was included in the binding incubation with prey.

### Analytical size exclusion chromatography

A Superose 6 Increase 3.2/300 column was equilibrated with buffer (25 mM HEPES-KOH pH 7.6, 100 mM sodium acetate, 0.5 mM TCEP, 2 mM EDTA). Ten micrograms of proteins at the same molarity (> 1 mM) was incubated together or separately in the above buffer at 30 °C for 5 min with shaking, before loading. Thirty microliter fractions were collected and analyzed by polyacrylamide gel electrophoresis and immunoblotting.

### Protein crosslinking mass spectrometry

Cohesin and Tof1-Csm3 were purified as above, but the buffer used during the final size exclusion chromatography step was 25 mM HEPES-KOH pH 7.6, 150 mM sodium acetate, 0.5 mM TCEP, and 2 mM EDTA. The final buffer for Chl1 was 25 mM HEPES-KOH pH 7.6, 100 mM NaCl, 0.5 mM TCEP, and 2 mM EDTA. To prepare Mrc1, 25 mM HEPES-KOH pH 7.6, 150 mM NaCl, 0.5 mM TCEP, and 2 mM EDTA were used during the final glycerol dialysis.

A total of 100 μg cohesin, Tof1-Csm3, and Chl1 with or without Mrc1 at 300–350 nM concentration was incubated at 30 °C for 5 min with shaking. The crosslinker Sulfo-SDA was added at a protein to crosslinker weight ratio of 1:0.75, and the mixtures were left in the dark at 4 °C for 2 h. The diazirine group in SDA was then photoactivated by UV irradiation at 365 nm from an CL-1000 Ultraviolet Crosslinker (Spectrum). Samples were spread as thin droplets on opened lids of Eppendorf tubes and placed on ice at a distance of 5 cm from the UV-A lamp and irradiated for 20 min. Unreacted NHS ester was then quenched with 20 mM ammonium bicarbonate at room temperature for 20 min. The proteins were precipitated with 4 volumes of acetone overnight at − 20 °C and protein pellets processed for mass spectrometry analysis.

Precipitated protein samples were resolubilized in digestion buffer (8M urea in 100 mM ammonium bicarbonate) to an estimated protein concentration of 1 mg/mL. Dissolved protein sample was reduced by addition of 1 M dithiothreitol (DTT) to a final concentration of 5 mM. The reaction was incubated at room temperature for 30 min. The free sulfhydryl groups in the sample were then alkylated by adding 500 mM iodoacetamide (final concentration of 15 mM) and incubation at room temperature for 20 min in the dark. After alkylation, additional DTT was added to a total concentration of 10 mM to quench excess of iodoacetamide. Next, protein samples were digested with LysC (at a 50:1 (m/m) protein to protease ratio) at room temperature for 4 h. The sample was then diluted with 100 mM ammonium bicarbonate to reach a urea concentration of 1.5 M. Trypsin was added at a 50:1 (m/m) protein to protease ratio to further digest proteins overnight (~ 15 h) at room temperature. Resulting peptides were desalted using C18 StageTips (Rappsilber et al. 2007).

For each sample, resulting peptides were fractionated using size exclusion chromatography to enrich for crosslinked peptides (Leitner et al. 2013). Peptides were separated using a Superdex™ 30 Increase 3.2/300 column (Cytiva) at a flow rate of 10 mL/min. The mobile phase consisted of 30% (v/v) acetonitrile and 0.1% trifluoroacetic acid. The earliest six peptide-containing fractions (50 μL each) were collected. Solvent was removed using a vacuum concentrator. The fractions were then analyzed by LC-MS/MS.

LC-MS/MS analysis was performed using an Orbitrap Fusion Lumos Tribrid mass spectrometer (Thermo Fisher Scientific), connected to an Ultimate 3000 RSLCnano system (Dionex, Thermo Fisher Scientific). Each size exclusion chromatography (SEC) fraction was resuspended in 1.6% v/v acetonitrile 0.1% v/v formic acid, and each SEC fraction was analyzed with duplicated LC-MS/MS acquisitions. Peptides were injected onto a 50-cm EASY-Spray C18 LC column (Thermo Scientific) that is operated at 50 °C column temperature. Mobile phase A consists of water and 0.1% v/v formic acid, and mobile phase B consists of 80% v/v acetonitrile and 0.1% v/v formic acid. Peptides were loaded and separated at a flowrate of 0.3 μL/min. Peptides were separated by applying a gradient ranging from 2 to 45% B over 90 min. The gradient was optimized for each fraction. Following the separating gradient, the content of B was ramped to 55% and 95% within 2.5 min each. Eluted peptides were ionized by an EASY-Spray source (Thermo Scientific) and introduced directly into the mass spectrometer.

MS data was acquired in the data-dependent mode with the top-speed option. For each 3-s acquisition cycle, the full scan mass spectrum was recorded in the Orbitrap with a resolution of 120,000. The ions with a charge state from 3+ to 7+ were isolated and fragmented using higher-energy collisional dissociation (HCD). For each isolated precursor, stepped collision energy (26%, 28%, or 30%) was applied. The fragmentation spectra were then recorded in the Orbitrap with a resolution of 50,000. Dynamic exclusion was enabled with single repeat count and 60-s exclusion duration.

The data from the two samples were processed separately. MS2 peak lists were generated from the raw mass spectrometry data files using the MSConvert module in ProteoWizard (v3.0.11729). Precursor and fragment m/z values were recalibrated. Identification of crosslinked peptides was carried out using xiSEARCH software (v1.7.6.4) (Mendes et al. 2019). The peak lists were searched against the sequences and the reversed sequences of the cohesin subunits and cohesion establishment factors present in the sample. The following parameters were applied for the search: MS accuracy = 3 ppm; MS2 accuracy = 5 ppm; enzyme = trypsin (with full tryptic specificity); allowed number of missed cleavages = 3; missing monoisotopic peak = 2; crosslinker = SDA (the reaction specificity for SDA was assumed to be for lysine, serine, threonine, tyrosine, and protein N termini on the NHS ester end, and any amino acids for the diazirine end); fixed modifications = carbamidomethylation on cysteine; variable modifications = oxidation on methionine, SDA loop link, hydrolyzed SDA on the diazirine end, acetylation on lysine, deamidation on asparagine, phosphorylation on serine, acetone modification on lysine and histidine; and maximum variable modification per peptide = 2.

Identified crosslinked peptide candidates that had at least three matched fragment ions (at least two containing crosslinked residue) in each crosslinked peptide were filtered using xiFDR (Fischer and Rappsilber 2018). A false discovery rate of 1% on residue-pair level was applied with “boost between” option selected. Finally, identified crosslinks from both analyzed samples were merged.

## Supplementary information

ESM 1: (PDF 3705 kb)

## Data Availability

The protein crosslink mass spectrometry data reported in this study has been deposited with the Japan Proteome Standard Repository jPOST (project ID JPST002006, PRIDE ID: PXD039609). Any unique reagents generated in this study are available from the authors upon reasonable request.
