# Supplementary material for: Replisome-cohesin interactions provided by the Tof1-Csm3 and Mrc1 cohesion establishment factors
Source: Chromosoma. 2023 May 11;132(2):117–35. doi: 10.1007/s00412-023-00797-4 (PMC10247859; doi:10.1007/s00412-023-00797-4)
Supplement: Supplementary file 1 — (PDF 3705 kb) [file 412_2023_797_MOESM1_ESM.pdf]

# Replisome-cohesin interactions provided by the Tof1-Csm3 and Mrc1 cohesion establishment factors

Sudikchya Shrestha, Masashi Minamino, Zhuo A. Chen, Céline Bouchoux, Juri Rappsilber, Frank Uhlmann

## Supplementary Information


| | Page |
|---|---|
| Supplementary Figures S1 – S8 | 2 |
| Table S1 Yeast strains used in this study | 12 |
| Table S2 Plasmids used in this study | 15 |
| Table S3 Antibodies used in this study | 16 |

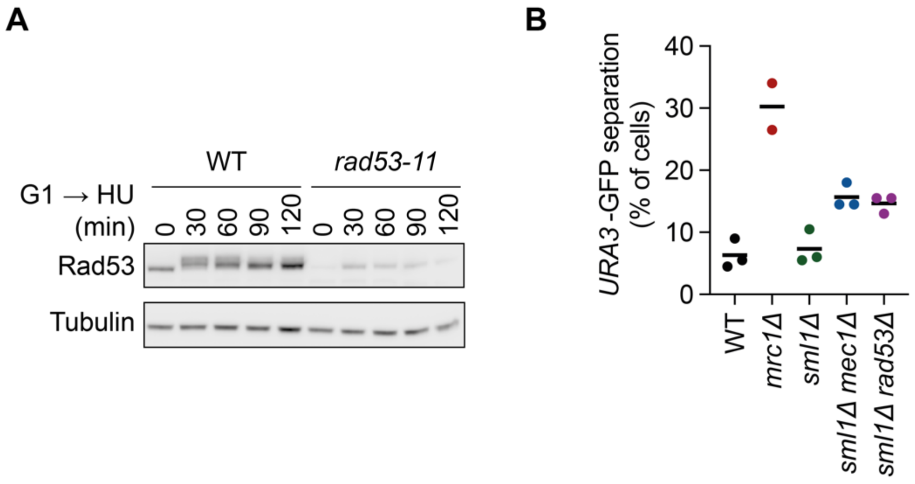


**Fig. S1** Additional experiments on the replication checkpoint. **A** Rad53 protein levels and electrophoretic mobility shift due to phosphorylation were analyzed by immunoblotting in wild type and *rad53-11* cells, synchronized in G1 and released into 100 mM hydroxyurea-containing medium. Tubulin served as a loading control. **B** Results from a GFP dot assay to monitor sister chromatid cohesion in strains of the indicated genotypes. The means and individual values from three independent experiments are shown. The absence of Mec1 or Rad53 from *sml1Δ* cells caused a statistically significant cohesion defect (unpaired *t*-tests, $p = 0.013$ and $p = 0.015$, respectively), that was however smaller than observed in *mrc1Δ* cells.

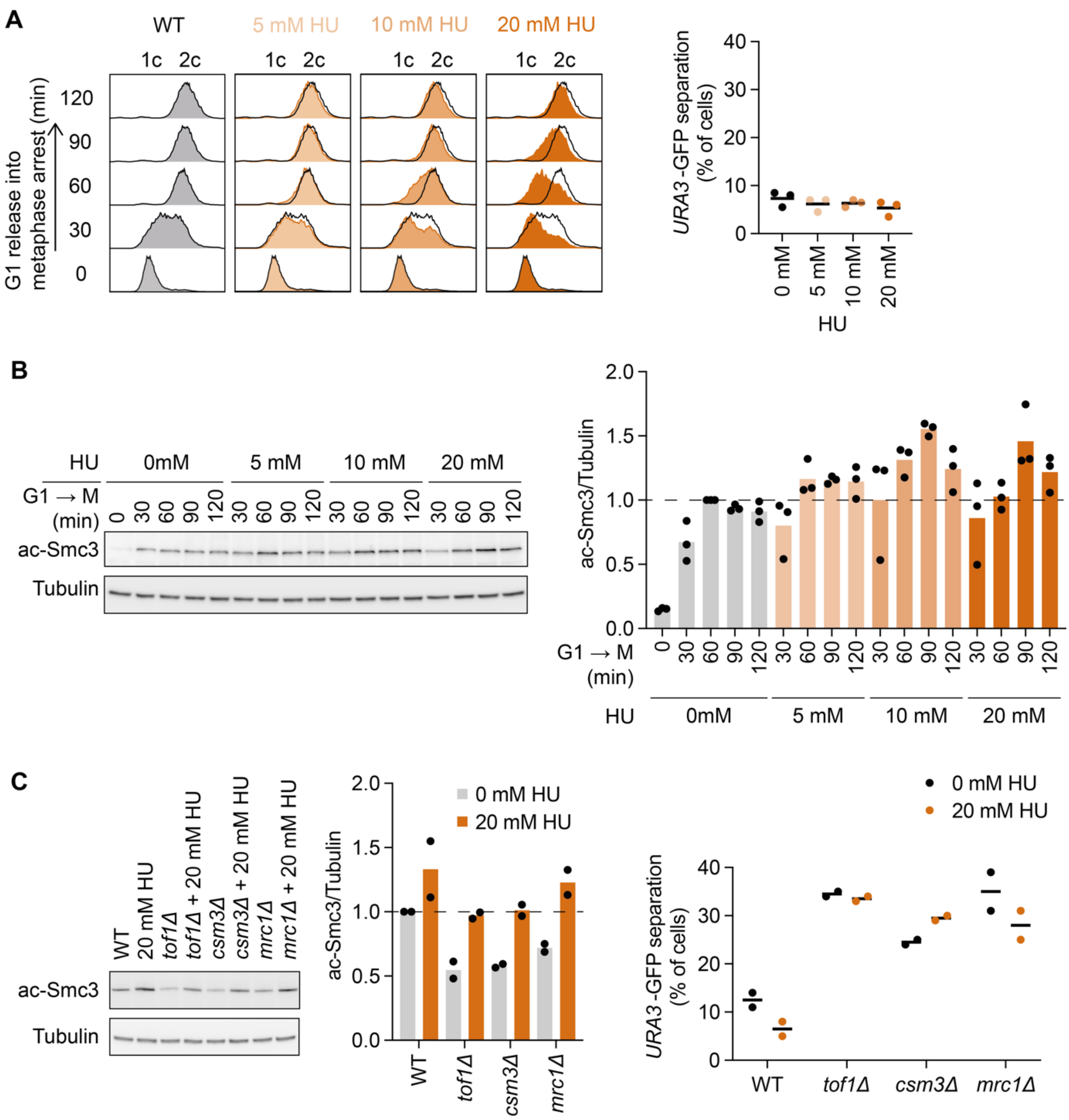


**Fig. S2** Hydroxyurea and cohesion establishment. **A** FACS analysis of DNA content of wild type cells following G1 release into medium containing nocodazole and the indicated HU concentrations. The profile without HU is overlaid onto the others as a dark grey outline. Sister chromatid cohesion was assessed by the GFP dot assay at the 120 minutes time point. Means and individual values of three independent repeat experiments are shown. Unpaired *t*-tests revealed no significant difference between control (0 mM HU) and any of the HU treated cells. **B** Smc3 acetylation during the same experiment was analyzed by immunoblotting. Tubulin served as a loading control. The ac-Smc3/tubulin ratios were normalized to the highest ratio observed in wild type cells. Means and individual values from three independent repeat

experiments are shown. Two-way ANOVA tests revealed significantly increased Smc3 acetylation in 10 mM HU treated cells ($p = 0.022$), while the differences remained insignificant at $p < 0.05$ at other concentrations. **C** Hydroxyurea restores Smc3 acetylation, but not sister chromatic cohesion, in cells lacking Tof1-Csm3 or Mrc1. Smc3 acetylation and sister chromatid cohesion were assessed in the strains of the indicated genotypes, 120 minutes after release from G1 arrest into medium containing nocodazole, with or without 20 mM HU. Means and individual values from two independent repeat experiments are shown.

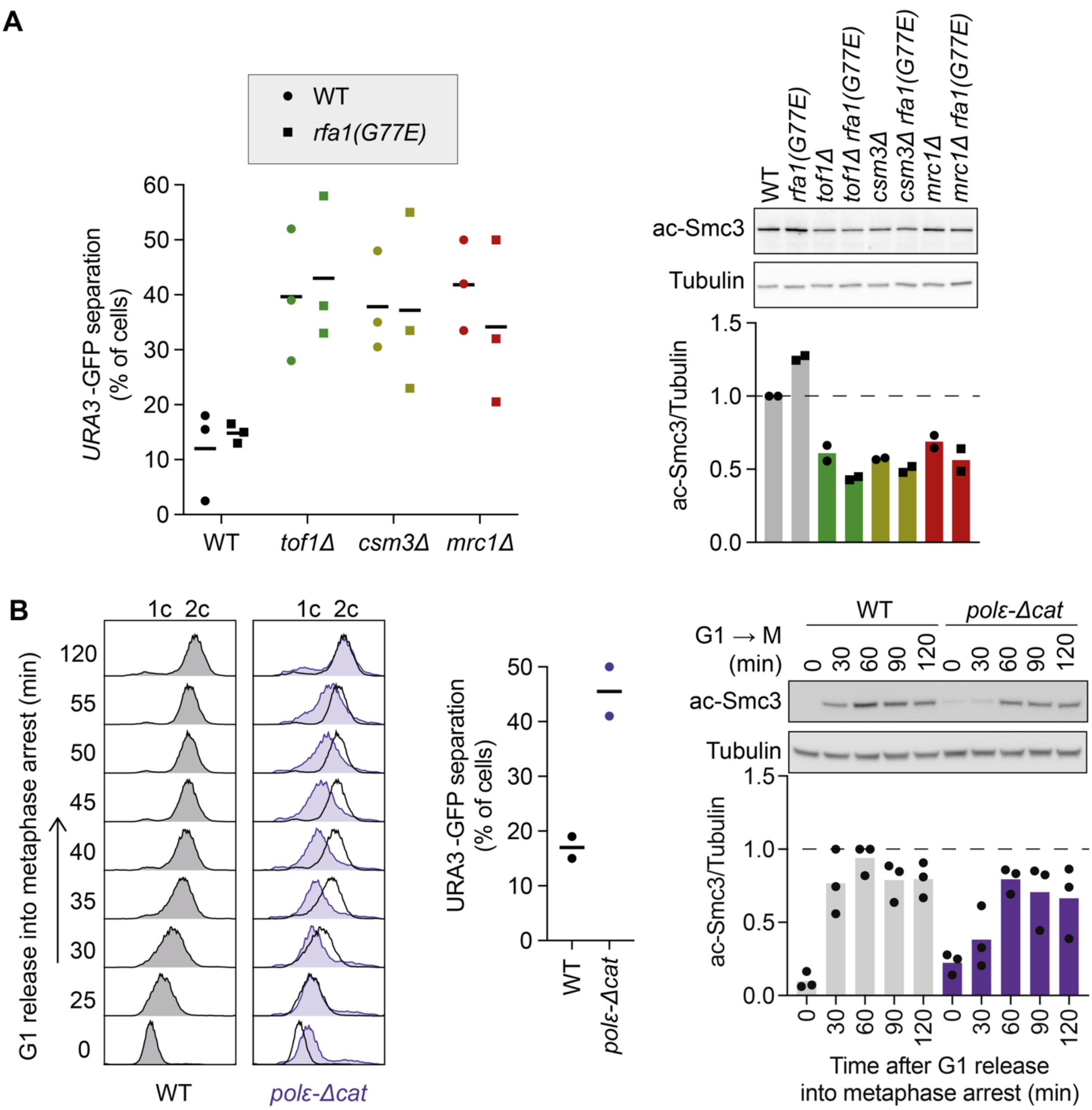


**Fig. S3** Additional experiments on helicase speed. **A** The *rfa1(G77E)* allele does not improve cohesion in cells lacking Tof1-Csm3 or Mrc1. Sister chromatid cohesion was analyzed 120 minutes after release of cells of the indicated genotypes from G1 block into nocodazole-imposed mitotic arrest. Means and individual values from three independent repeat experiments are shown. Unpaired *t*-tests revealed no significant differences between WT and *rfa1(G77E)* backgrounds in all cases. (We earlier reported defective cohesion in *rfa1(G77E)-HA*$_3$ cells (Murayama et al. 2018). Further investigations revealed that the defect resulted from the HA epitope tag, not the *rfa1(G77E)* mutation). We also found no noticeable rescue of cohesin acetylation by the *rfa1(G77E)* allele in two independent repeat experiments. The individual values and means of the quantified Smc3 acetylation signals are shown. **B** A cohesion

defect in cells lacking the DNA polymerase ε catalytic domain (*polε-Δcat*). Flow cytometry profiles of wild type and *polε-Δcat* cells after release from G1 block into nocodazole-imposed mitotic arrest. The wild type profile is overlaid onto the *polε-Δcat* profile as a dark grey outline. Sister chromatid cohesion was analyzed at 120 minutes. The individual values and means from two independent repeat experiments are shown. Despite of the strong sister chromatid cohesion defect, Smc3 acetylation was only mildly affected. The means and individual Smc3 acetylation values from three independent repeat experiments are shown. A two-way ANOVA test revealed that the difference between wt and *polε-Δcat* cells was insignificant at $p < 0.05$.

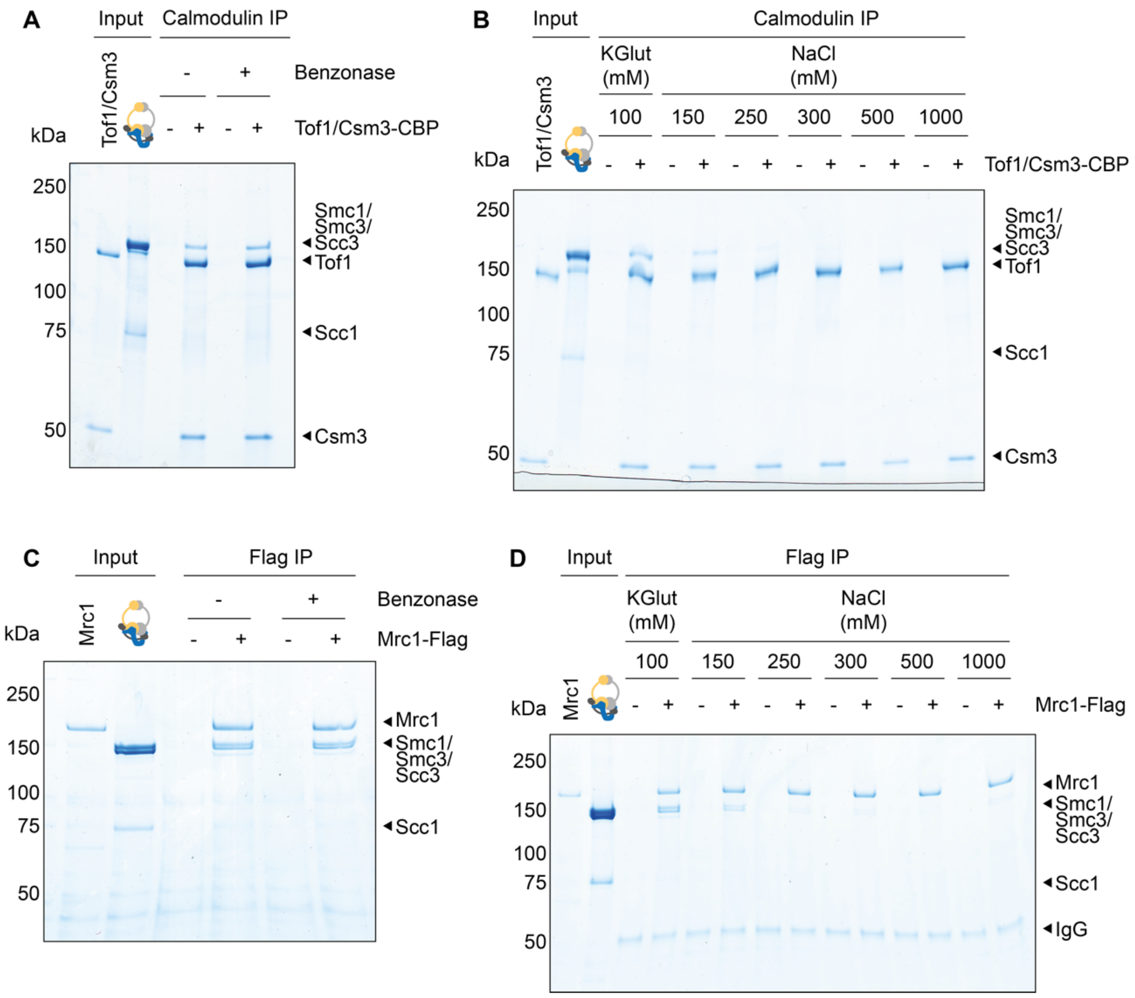


**Fig. S4** Protein interaction control experiments. **A** Interaction screen with Tof1-Csm3 as the bait. Tof1-Csm3-coated or control beads were incubated with cohesin in the presence or absence of benzonase, or **B** in the presence of increasing NaCl concentrations. 10% of input proteins are loaded next to the bead-bound fractions. Proteins were visualized by Coomassie Blue staining. **C** and **D** As above, but Mrc1 served as the bait.

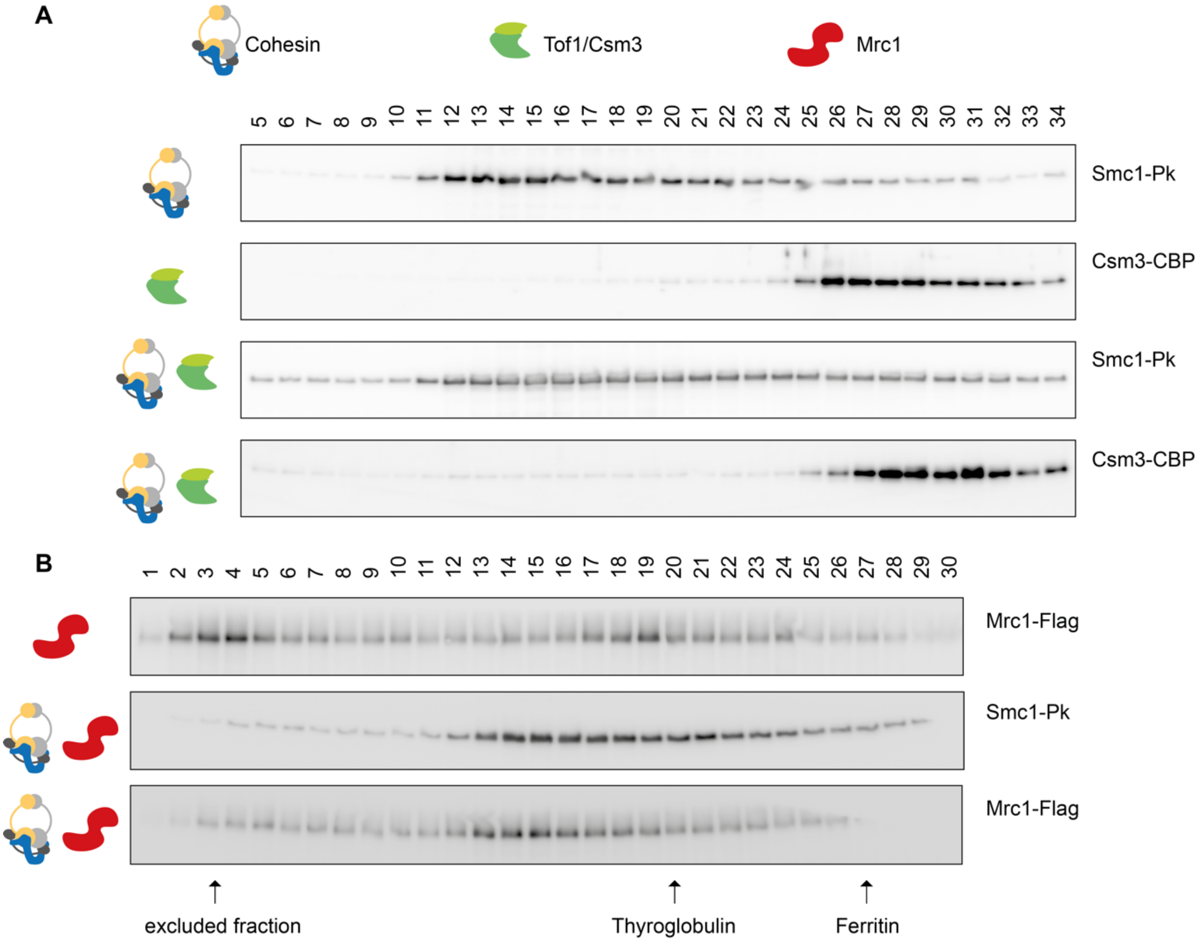


**Fig. S5** Protein interaction analysis between cohesin and Tof1-Csm3 or Mrc1 by size exclusion chromatography. **A** Cohesin and Tof1-Csm3 were incubated separately or in combination, then loaded onto a Superose 6 size exclusion column. Eluted fractions were analyzed by immunoblotting. The excluded and elution volumes of size markers are indicated. **B** As above, but cohesin and Mrc1 were analyzed.

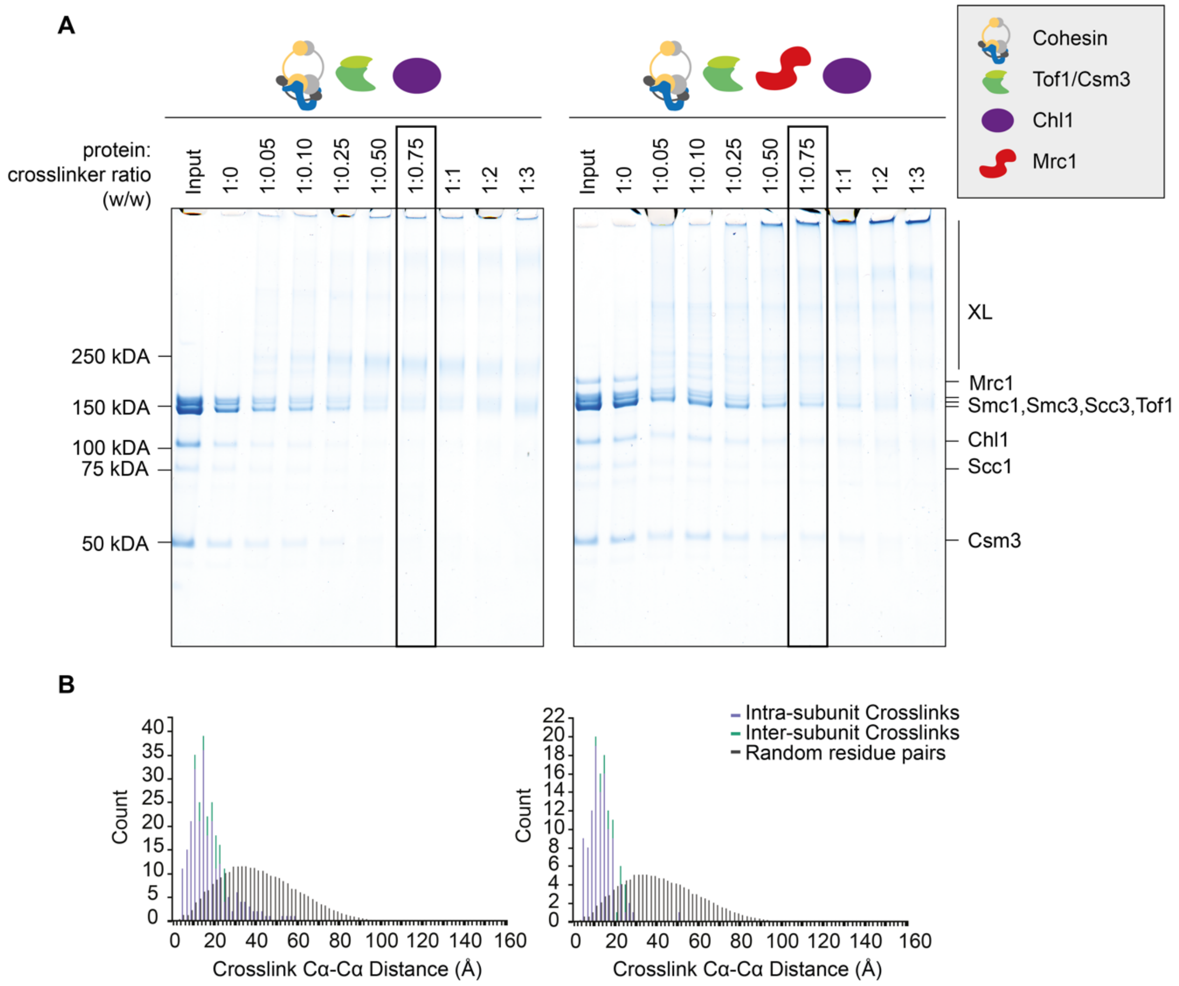


**Fig. S6** Controls accompanying the CLMS experiment. **A** Sulfo-SDA crosslinker titration. Cohesin and the indicated cohesion establishment factors were incubated with the indicated Sulfo-SDA concentrations. Crosslinked samples were analyzed by SDS-polyacrylamide gel electrophoresis followed by Coomassie Blue staining. Based on this analysis, a protein : crosslinker ratio of 1 : 0.75 was selected to prepare the samples for mass spectrometry analysis. **B** Histograms of Cα-Cα distances of intra- (purple) and intersubunit (green) crosslinks detected within Tof1-Csm3, measured on the Tof1-Csm3 cryo-EM structure (PDB: 6SKL). Distances between a sampling of random residue pairs in the structure are provided in grey. Similar histograms of Cα-Cα distances were also obtained for the various structured parts of the cohesin complex. In each case, the median crosslink distance was 8-12 Å with the majority of linkages contained within the theoretical crosslinking limit of 25 Å.

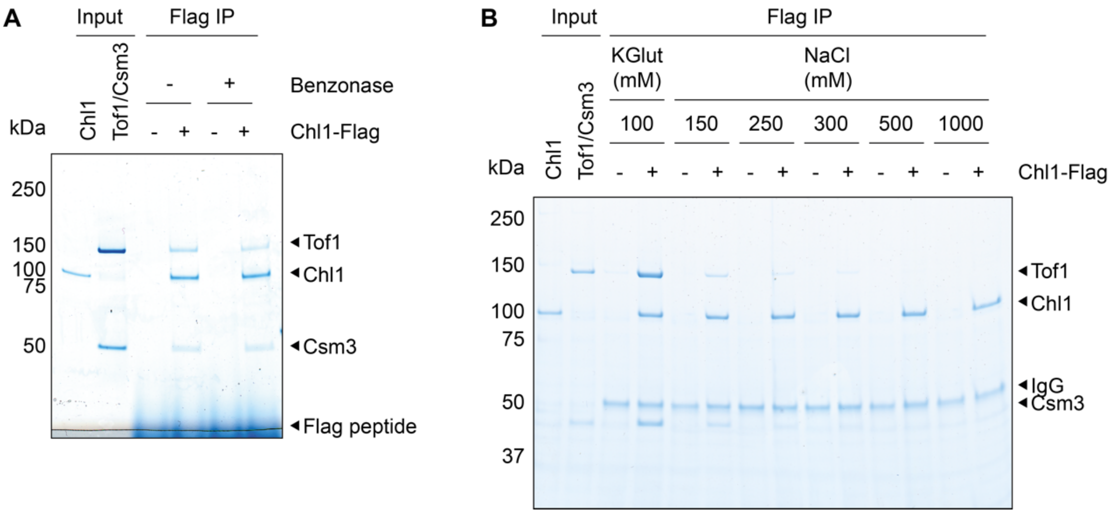


**Fig. S7** Tof1-Csm3 interacts with Chl1. **A** Interaction screen with Chl1 as the bait. Chl1-coated or control beads were incubated with Tof1-Csm3, in the absence or presence of benzonase. 10% of input proteins are loaded next to the bead-bound fractions. Proteins were visualized by Coomassie Blue staining. **B** As above, but increasing concentrations of NaCl were included in the incubation.

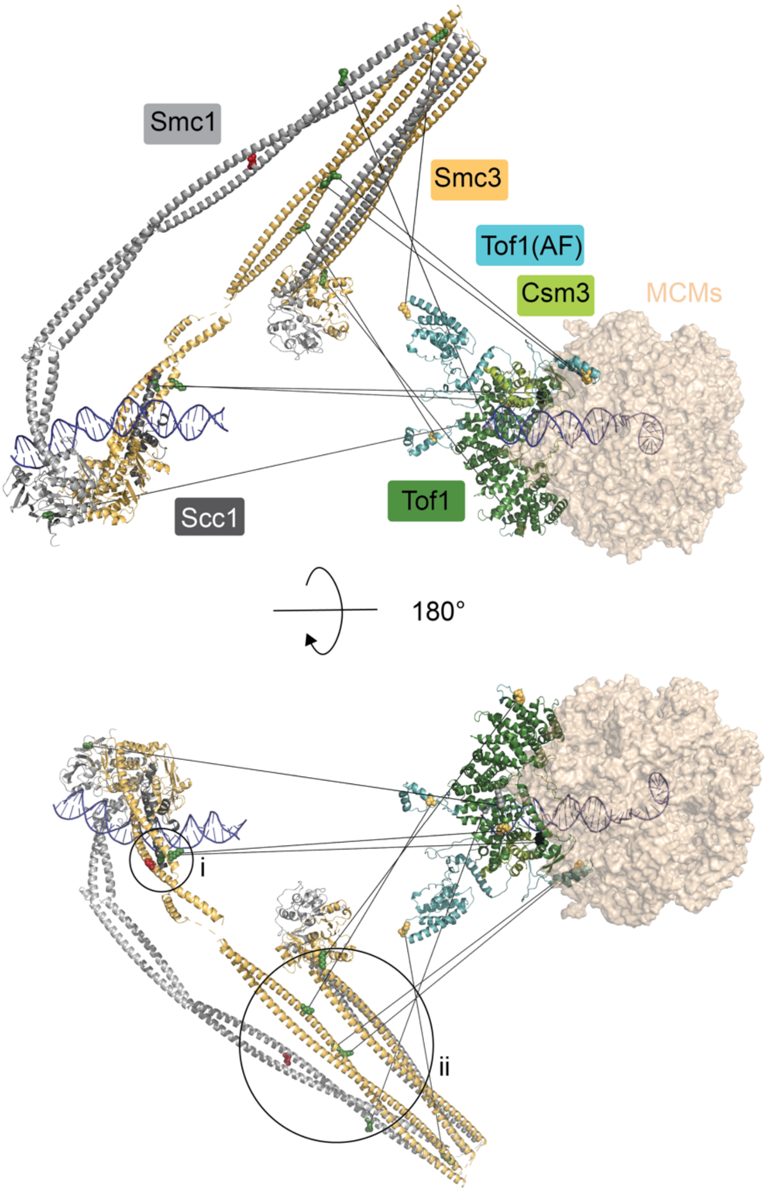


**Fig. S8** Composite structural model of Tof1-Csm3 encountering cohesin. Models of the Smc1-Smc3 dimer (Fig. 6B) and of Tof1-Csm3 (Fig. 7A) were placed next to each other. Crosslinks are indicated with black lines. Circles highlight linkage site clusters (i) at the Smc3 joint, location of the kleisin N-gate, and (ii) along the Smc1-Smc3 coiled coils, respectively.

## *Table S1 Yeast strains used in this study*

| Strain | Genotype (W303 background) | Source |
|---|---|---|
| Y193 | *MAT* a *ura3::3XURA3 tetO$_{112}$, his3::HIS3tetR-GFP* | Lab stock |
| Y194 | *MAT* α *ura3::3XURA3 tetO$_{112}$, his3::HIS3tetR-GFP* | Lab stock |
| Y3854 | *MAT* **a** *ura3::3XURA3 tetO$_{112}$, his3::HIS3tetR-GFP tof1Δ::TRP1* | Lab stock |
| Y3855 | *MAT* **a** *ura3::3XURA3 tetO$_{112}$, his3::HIS3tetR-GFP csm3Δ::TRP1* | Lab stock |
| Y3857 | *MAT* **a** *ura3::3XURA3 tetO$_{112}$, his3::HIS3tetR-GFP mrc1Δ::TRP1* | Lab stock |
| PP37/Y6804 | *MAT* **a** *RAD5, ura3::URA3/GPD-TK7, rad53-11* | (Forey et al., 2020) |
| Y6802 | *MAT* **a** *ura3::3XURA3 tetO$_{112}$, his3::HIS3tetR-GFP rad53-11* | This study |
| Y6644 | *MAT* **a** *ura3::3XURA3 tetO$_{112}$, his3::HIS3tetR-GFP mrc1Δ::TRP, mrc1$^{AQ}$-myc9::LEU2* | This study |
| Y6805 | *MAT* **a** *ura3::3XURA3 tetO$_{112}$, his3::HIS3tetR-GFP mrc1ΔC$^{(Δ844-1096)}$-myc9::TRP1* | This study |
| Y6806 | *MAT* **a** *ura3::3XURA3 tetO$_{112}$, his3::HIS3tetR-GFP MRC1-myc9::TRP1* | This study |
| Y5770 | *MAT* **a** *ura3::3XURA3 tetO$_{112}$, his3::HIS3tetR-GFP sml1Δ::TRP1* | This Study |
| Y1092 | *MAT* **a** *ura3::3XURA3 tetO$_{112}$, his3::HIS3tetR-GFP sml1Δ::URA3, mec1Δ::TRP1* | Lab stock |
| Y6807 | *MAT* **a** *ura3::3XURA3 tetO$_{112}$, his3::HIS3tetR-GFP sml1Δ::TRP1, rad53Δ::LEU2* | This study |
| Y5771 | *MAT* **a** *ura3::3XURA3 tetO$_{112}$, his3::HIS3tetR-GFP tof1Δ::TRP1, sml1Δ::LEU2* | This study |
| Y5772 | *MAT* **a** *ura3::3XURA3 tetO$_{112}$, his3::HIS3tetR-GFP* | This study |

| | | |
|---|---|---|
| | *csm3Δ::TRP1, sml1Δ::LEU2* | |
| Y5773 | *MAT* **a** *ura3::3XURA3 tetO*$_{112}$*, his3::HIS3tetR-GFP mrc1Δ::TRP1, sml1Δ::LEU2* | This study |
| Y6733 | *MAT* **a** *ura3::3XURA3 tetO*$_{112}$*, his3::HIS3tetR-GFP MRC1-Flag3::NAT*$^R$ | This study |
| Y6734 | *MAT* **a** *ura3::3XURA3 tetO*$_{112}$*, his3::HIS3tetR-GFP MRC1 8D-Flag3::NAT*$^R$ | This study |
| Y6808 | *MAT* **a** *ura3::3XURA3 tetO*$_{112}$*, his3::HIS3tetR-GFP polε-Δcat::TRP1* | This study |
| Y6182 | *MAT* **a** *ura3::3XURA3 tetO*$_{112}$*, his3::HIS3tetR-GFP tof1ΔC(Δ982-1238)-HA3::TRP1* | This study |
| Y6185 | *MAT* **a** *ura3::3XURA3 tetO*$_{112}$*, his3::HIS3tetR-GFP TOF1-HA3::TRP1* | This study |
| Y6732 | *MAT* **a** *ura3::3XURA3 tetO*$_{112}$*, his3::HIS3tetR-GFP top1Δ::TRP1* | This study |
| Y6179 | *MAT* **a** *ura3::3XURA3 tetO*$_{112}$*, his3::HIS3tetR-GFP tof1(Δ628-1238)-HA3::TRP1* | This study |
| Y6180 | *MAT* **a** *ura3::3XURA3 tetO*$_{112}$*, his3::HIS3tetR-GFP tof1(Δ763-1238)-HA3::TRP1* | This study |
| Y6181 | *MAT* **a** *ura3::3XURA3 tetO*$_{112}$*, his3::HIS3tetR-GFP tof1(Δ831-1238)-HA3::TRP1* | This study |
| Y6104 | *MAT* **a** *ura3::3XURA3 tetO112, his3::HIS3tetR-GFP, rfa1(G77E)::KAN*$^R$ | This study |
| Y6809 | *MAT* **a** *ura3::3XURA3 tetO112, his3::HIS3tetR-GFP, rfa1(G77E)::KAN*$^R$*, tof1Δ::LEU2* | This study |
| Y6810 | *MAT* **a** *ura3::3XURA3 tetO112, his3::HIS3tetR-GFP, rfa1(G77E)::KAN*$^R$*, csm3Δ::LEU2* | This study |
| Y6811 | *MAT* **a** *ura3::3XURA3 tetO112, his3::HIS3tetR-GFP, rfa1(G77E)::KAN*$^R$*, mrc1Δ::LEU2* | This study |

| | | |
|---|---|---|
| Y5226 | *MAT* **a** *Δpep4::HIS3 Δwpl1::LEU2 Δeco1::KanMX*<br>*pGAL (GAL4 + SMC1-Pk3 + SMC3 + SCC1-3C- ProtA + SCC3-myc)* | Lab Stock |
| YAE48 | MAT **a** *bar1::Hyg$^R$ pep4::KanMX*<br>*ura3::URA3pRS306-CBP-CSM3 + Tof1* | Diffley Lab Stock |
| YJY32 | MAT **a** *bar1::Hyg$^R$ pep4::KanMX*<br>*his3::HIS3pRS303-MRC1-Flag2* | Diffley Lab Stock |
| Y5562 | *MAT* **a** *Δpep4::HIS3, pGAL1 CHL1-Flag-2ProtA,* | Lab Stock (MM) |
| Y4702 | *MAT* **a** *Δpep4::HIS3, Δwpl1::LEU2, Δeco1::KanMX,*<br>*pRSII402-pGAL1-10 GAL4 SMC1-Pk-2ProtA*<br>*YIplac204-pGAL1-SMC3-HA3* | Lab Stock (CB) |
| Y4703 | *MAT* **a** *Δpep4::HIS3, Δwpl1::LEU2, Δeco1::KanMX,*<br>*pRSII402-pGAL1-10 GAL4 SMC1-Pk3*<br>*YIplac204-pGAL1-10 SMC3-HA3 SCC1-HIS-HA-3C-2ProtA* | Lab Stock (CB) |

## *Table S2. Plasmids used in this study*

| Plasmid Number | Description | Source |
|---|---|---|
| pD2043 | pRS305-pMRC1-mrc1(AQ) | Diffley Lab (AM) |
| p1942 | pRS305-pMRC1-mrc1(AQ)-myc9 | This Study |
| pD3092 | pRS40N-MRC1-Flag3 | Diffley Lab (AM) |
| pD3093 | pRS40N-MRC1(8D)-Flag3 | Diffley Lab (AM) |
| p2036 | YIplac204-pPOL2(371 bp)-POL2(1263-1797)-prePOL(526-326 bp upstream) | This Study |

***Table S3 Antibodies used in this study***

| Protein detected | Biological source and clonality | Source | Dilution |
|---|---|---|---|
| α-ac-Smc3 | mouse monoclonal | gift from the Shirahige lab | 1 : 500 |
| α-Tubulin | mouse monoclonal clone TAT-1 | Cell Services Science Technology Platform, The Francis Crick Institute | 1 : 5000 |
| α-Rad53 | rabbit polyclonal | Abcam (ab104232) | 1 : 2000 |
| α-Rad53 | mouse monoclonal | Abcam (ab166859) | 1 : 2000 |
| α-Myc | mouse monoclonal clone 9E10 | Santa Cruz Biotechnology | 1 : 2000 |
| α-Flag | mouse monoclonal clone M2 | Merck (F3165) | 1 : 2000 |
| α-HA | mouse monoclonal clone 12CA5 | Cell Services Science Technology Platform, The Francis Crick Institute | 1 : 2000 |
| α-HA | rabbit polyclonal | Abcam (ab9110) | 1 : 2000 |
| α-Sml1 | rabbit polyclonal | Antibodies-online GmbH (ABIN3197499) | 1 in 1000 |
| α-Rfa1 | rabbit polyclonal | Antibodies-online GmbH (ABIN4369396) | 1 in 5000 |
| α-RNR1 | rabbit polyclonal | Antibodies-online GmbH (ABIN4966296) | 1 in 5000 |
| α-CBP | rabbit monoclonal clone C16T | Merck (04-932) | 1 in 2000 |
| α-CBP | rabbit polyclonal | Merck (07-482) | 1 in 2000 |
| α-Pk(V5) | mouse monoclonal clone SV5-Pk1 | BioRad (MCA1360) | 1 in 2000 |
| α-myc | mouse monoclonal clone 9B11 | Cell Signalling Technology (2276) | 1 in 2000 |
| α-myc | goat polyclonal | Abcam (ab9132) | 1 in 2000 |
| α-Pk(V5) | rabbit polyclonal | Abcam (ab15828) | 1 in 2000 |
